# Structural‐Activity Relationship‐Inspired the Discovery of Saturated Fatty Acids as Novel Colistin Enhancers

**DOI:** 10.1002/advs.202302182

**Published:** 2023-08-08

**Authors:** Jinju Cai, Jingru Shi, Chen Chen, Mengping He, Zhiqiang Wang, Yuan Liu

**Affiliations:** ^1^ Jiangsu Co‐innovation Center for Prevention and Control of Important Animal Infectious Diseases and Zoonoses College of Veterinary Medicine Yangzhou University Yangzhou 225009 China; ^2^ Joint International Research Laboratory of Agriculture and Agri‐Product Safety the Ministry of Education of China Yangzhou University Yangzhou 225009 China; ^3^ Institute of Comparative Medicine Yangzhou University Yangzhou 225009 China

**Keywords:** antibiotic adjuvant, antimicrobial resistance, colistin, MCR

## Abstract

The emergence and prevalence of mobile colistin resistance gene *mcr* have dramatically compromised the clinical efficacy of colistin, a cyclopeptide antibiotic considered to be the last option for treating different‐to‐treat infections. The combination strategy provides a productive and cost‐effective strategy to expand the lifespan of existing antibiotics. Structural‐activity relationship analysis of polymyxins indicates that the fatty acyl chain plays an indispensable role in their antibacterial activity. Herein, it is revealed that three saturated fatty acids (SFAs), especially sodium caprate (SC), substantially potentiate the antibacterial activity of colistin against *mcr‐*positive bacteria. The combination of SFAs and colistin effectively inhibits biofilm formation and eliminates matured biofilms, and is capable of preventing the emergence and spread of mobile colistin resistance. Mechanistically, the addition of SFAs reduces lipopolysaccharide (LPS) modification by simultaneously promoting LPS biosynthesis and inhibiting the activity of MCR enzyme, enhance bacterial membrane damage, and impair the proton motive force‐dependent efflux pump, thereby boosting the action of colistin. In three animal models of infection by *mcr*‐positive pathogens, SC combined with colistin exhibit an excellent therapeutic effect. These findings indicate the therapeutic potential of SFAs as novel antibiotic adjuvants for the treatment of infections caused by multidrug‐resistant bacteria in combination with colistin.

## Graphical abstract

Inspired by the structure‐activity relationship of polymyxins, this work reports that three saturated fatty acids (SFAs), particularly sodium caprate, effectively boost the antibacterial activity of colistin against *mcr*‐positive bacteria both in vitro and in vivo. Notably, the addition of SFAs reduce lipopolysaccharide modification, enhance membrane damage and impair the function of efflux pump, thereby potentiating the action of colistin.

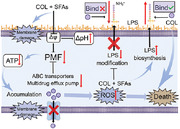

## 1 Introduction

The uncontrolled rise and spread of antimicrobial resistance has become a major global public health crisis,^[^ ^1^ ^]^ severely exacerbating the economic burden on healthcare systems.^[^ ^2^ ^]^ It also threatens human health, with antibiotic‐resistant bacteria predicted to cause 10 million deaths annually by 2050.^[^ ^3^ ^]^ Polymyxins (i.e., polymyxin B and colistin) are lipopeptide antibiotics that were approved in the late 1950s but were gradually abandoned for clinical use owing to their potential toxicities and the introduction of safer antibiotics.^[^ ^4^ ^]^ However, due to the rise of multidrug‐resistant (MDR) Gram‐negative pathogens, polymyxin antibiotics have been revived and are recognized as a last‐line option for the treatment of difficult‐to‐treat bacterial infections.^[^ ^5^ ^]^ The bactericidal activity of colistin is mainly dependent on the disruption of membrane permeability through the electrostatic interaction between positively‐charged residues of colistin and negatively charged lipid A moieties of lipopolysaccharide (LPS) anchored to the bacterial outer membrane.^[^ ^6^ ^]^ MCR‐1,^[^ ^7^ ^]^ a novel member of the phosphoethanolamine (pEtN) transferases family, transfers pEtN residues to lipid A in the outer membrane of Gram‐negative bacteria, resulting in a reduction in the negative charge in lipid A and a significant decrease in the affinity between colistin and LPS, thereby inducing colistin resistance.^[^ ^8^ ^]^ Until now, the plasmid‐borne *mcr‐1* gene and its variants have spread globally via horizontal gene transfer between intra‐ and inter‐species, leading to the emergence of MDR superbugs.^[^ ^9^ ^]^

To overcome *mcr*‐positive Gram‐negative pathogens, novel treatment strategies are urgently required. One strategy is to discover entirely new antibiotics, such as darobactin obtained from *Photorhabdus* isolates, which was active against important Gram‐negative pathogens by targeting BamA, an essential outer membrane protein. In addition, two analogues of colistin, including a naturally inspired antibiotic macolacin^[^ ^10^ ^]^ and a synthetic lipopeptide F365,^[^ ^11^ ^]^ displayed improved antibacterial activity against *mcr*‐carrying bacteria. Nevertheless, a new drug takes > 10 years from screening to market and costs an average $2.6 billion. Another more cost‐effective approach is to fully utilize existing antimicrobial agents, such as antibiotic adjuvant strategy.^[^ ^12^ ^]^

Antibiotic adjuvants are chemical compounds that increase bacterial susceptibility to antibiotics without altering the structure of the antibiotic.^[^ ^13^ ^]^ These compounds generally have no or only weak antibacterial activity, but when used in combination with antibiotics, they can restore or enhance the activity of commonly used antibiotics against drug‐resistant bacteria. It has the advantage of being low‐cost, safe, and efficient compared to developing new drugs. For example, clavulanic acid is the first β‐lactamase inhibitor to be introduced into the clinic, which protects the efficacy of antibiotics by inhibiting β‐lactamase activity and preventing its hydrolysis of antibiotics.^[^ ^14^ ^]^ In addition, it has been shown that even low doses of felodipine resensitized *Staphylococcus aureus* (MRSA) to aminoglycoside antibiotics.^[^ ^15^ ^]^ Diclofenac, a nonsteroidal anti‐inflammatory drug, restored the activity of β‐lactam antibiotics against methicillin‐resistant *S. aureus*.^[^ ^16^ ^]^ Also, some antibiotic adjuvants for colistin have been reported, for example, commercial artemisinin derivatives combined with colistin may prevent severe Gram‐negative bacterial infections.^[^ ^17^ ^]^ Melatonin, used for the treatment of sleep disorders and circadian rhythm disorders, can be effectively combined with colistin against pathogens carrying *mcr* gene.^[^ ^18^ ^]^ The above examples inspired us to search for antibiotic adjuvants from Food and Drug Administration (FDA)‐approved compounds as effective options for combating drug‐resistant pathogens. However, due to practical and technical limitations, no colistin adjuvants have been applied in clinical trials yet. Therefore, there is still an urgent need to explore safer and more effective colistin enhancers.

**Figure 1:**
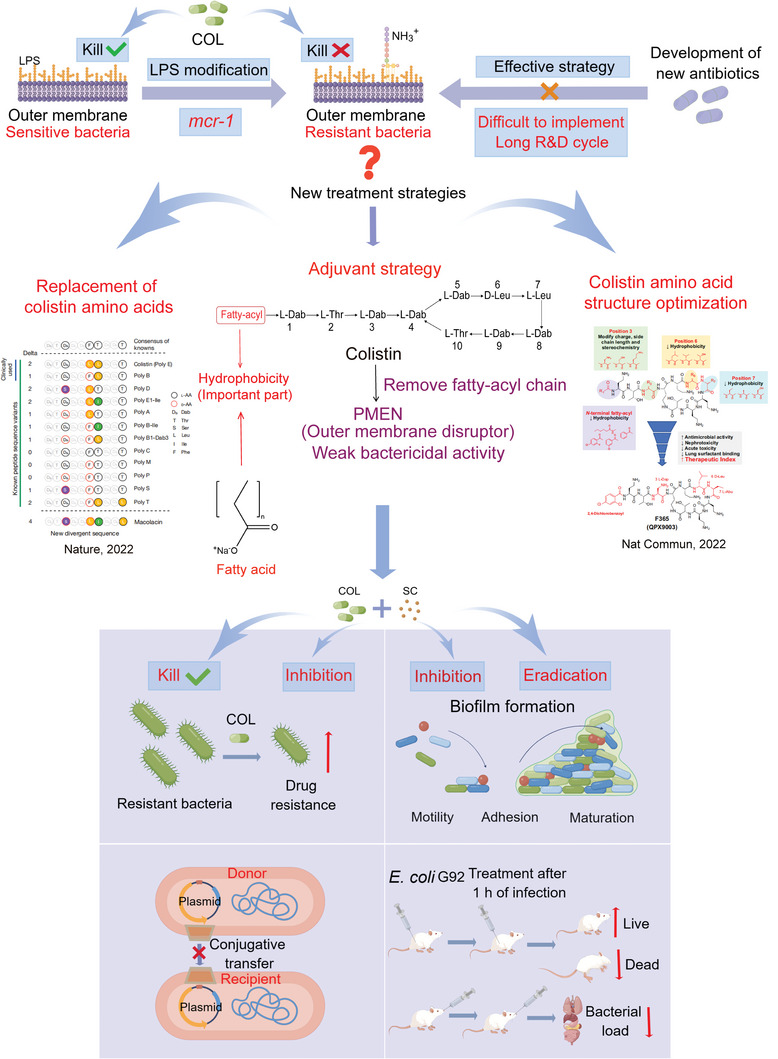
Schematic illustration of SFAs as novel antibiotic adjuvants for combating *mcr*‐positive Gram‐negative pathogens in combination with colistin (COL). Adapted with permission from ref. [10, 11] Copyright 2022 Springer Nature.

Colistin consists of a heptapeptide ring and a linear tripeptide side chain with an *N*‐terminal fatty acyl group. Among, the fatty acyl chain is typically a hydrophobic group, which facilitates the insertion of colistin into the bilayer of bacterial membranes, causing membrane disruption, ultimately leading to the leakage of bacterial contents and bacterial death. Consistently, the absence of fatty acyl chain would completely eliminate the antibacterial activity of colistin. For example, polymyxin B nonapeptide (PMBN), a cationic cyclic peptide derived from the naturally occurring peptide polymyxin B, is used as an outer membrane permeabilizer and has no direct antibacterial activity.^[^ ^19^ ^]^ This evidence suggests the indispensable role of fatty acyl chain in polymyxins action. Fatty acids, the essential components of lipids in plants, animals, and microorganisms, possess versatile biological functions. For instance, lauric acid was an inhibitor of *Clostridium difficile* growth in vitro and reduced inflammation in a mouse infection model.^[^ ^20^ ^]^ Moreover, sodium caprate (SC) is approved by the FDA as a safe food additive for human consumption,^[^ ^21^ ^]^ and related studies have shown that it can promote the absorption of poorly permeable drugs by intestinal epithelial cells.^[^ ^22^ ^]^ Based on these points, we hypothesized whether the addition of SFAs could boost colistin activity against drug‐resistant bacteria. To test this hypothesis, in this study, we investigated the potential of a series of SFAs as novel colistin enhancers in tackling *mcr*‐positive bacterial infections and elucidated the underlying mechanisms of action (**Figure** **1**).

## 2 Results

### 2.1 Synergistic Activity of Saturated Fatty Acids (SFAs) and Colistin against *mcr*‐Positive Bacteria

**Figure 2:**
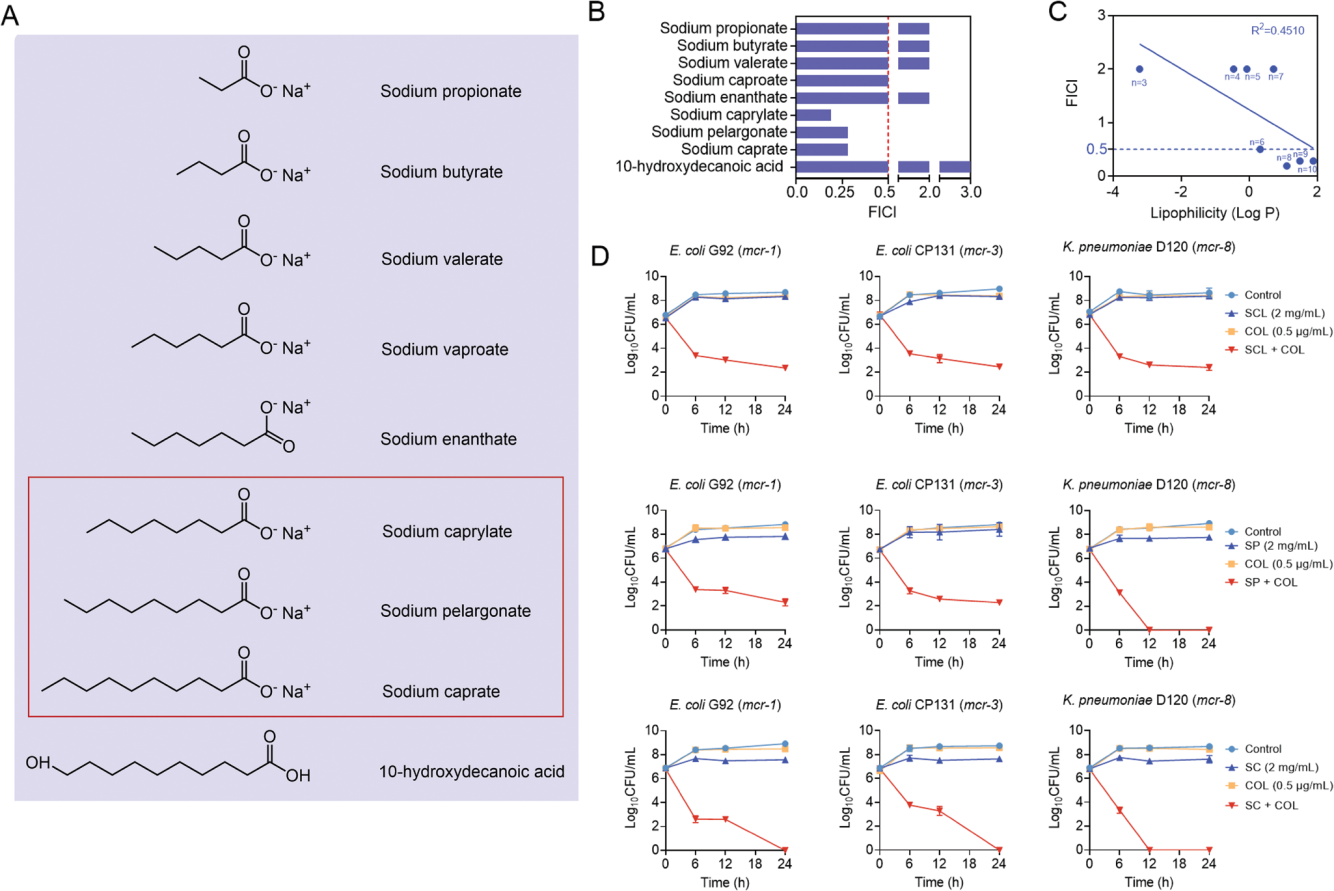
Synergistic activity between SFAs and colistin against *mcr*‐positive bacteria. A) Chemical structures of saturated fatty acids (SFAs). B) FICI values of colistin and SFAs with different carbon atom numbers against *E. coli* G92. C) Linear regression curve between FICI and Log P of SFAs. D) Time‐dependent killing curves of *E. coli* (*mcr‐1*), *E. coli* (*mcr‐3*), and *K. pneumoniae* (*mcr‐8*) treated by the three SFAs (SCL, SP, and SC at 2 mg mL^−1^) and colistin (0.5 µg mL^−1^) alone or their combination for 24 h. Data are representative of three independent experiments and shown as mean ± SD.

To evaluate the synergistic antibacterial activity of saturated fatty acids (SFAs) and colistin, we conducted a checkerboard assay between colistin and nine SFAs against *mcr*‐positive *Escherichia coli* (*E. coli*) G92, including sodium propionate (n = 3), sodium butyrate (n = 4), sodium valerate (n = 5), sodium caproate (n = 6), sodium enanthate (n = 7), sodium caprylate (n = 8), sodium pelargonate (n = 9), sodium caprate (n = 10), and 10‐hydroxydecanoic acid (n = 10, the 10th carbon atom is modified with ‐OH) (**Figure** **2**A). Interestingly, we found that three SFAs, including sodium caprylate (SCL), sodium pelargonate (SP), and sodium caprate (SC), enhanced the antibacterial activity of colistin against *E. coli* G92 (fractional inhibitory concentration index (FICI) < 0.5). Other SFAs had no obvious synergistic antibacterial activity with colistin, while 10‐hydroxydecanoic acid antagonized colistin activity (FICI > 3.0) (Figure 2B; Figure S1, Supporting Information). This may be because the modification of the hydroxyl group of the 10th carbon atom prevents the fatty acid from being inserted into the cell membrane. Furthermore, we continued to explore the relationship between the oil‐water partition coefficient (Log P) of tested SFAs and their synergistic activity. The linear regression curve of Log P and FICI values was shown in Figure 2C. Interestingly, there was a negative correlation between Log P and FICI, implying that as the increase of carbon atom number of SFAs, the Log P values gradually increase, while the FICI values gradually decrease, along with enhanced synergistic activity.

Then we tested their synergistic activity in important pathogens carrying various *mcr* variants. As expected, we also observed significant synergistic effects in *E. coli/Klebsiella pneumoniae* (*K. pneumoniae*) carrying *mcr‐3*/*mcr‐8*, and the minimal inhibition concentration (MIC) values of colistin were reduced by up to 32‐fold (Figure S2, Supporting Information). To study whether this effect is only effective for drug‐resistant bacteria, we tested their synergistic activity in engineered colistin‐resistant *E. coli* DH5α (pUC19‐*mcr‐1*) and susceptible bacteria *E. coli* ATCC 25922 and *E. coli* DH5α (pUC19). Interestingly, we found that three SFAs (SCL, SP and SC) and colistin showed potent synergistic activity against drug‐resistant bacteria, while no synergistic effect on sensitive bacteria, indicating the effect of SFAs is associated with the inhibition of specific drug resistance determinants (Figure S3, Supporting Information).

Next, we conducted a time‐killing curve to explore their synergistic bactericidal activity. We found that 1/4 MIC of colistin and 1/4 MIC of three SFAs (SCL, SP, and SC) had almost no bactericidal activity against drug‐resistant bacteria when used alone. By contrast, the combined use of SCL/SP and colistin dramatically reduced the loads of drug‐resistant bacteria by ≈ 4‐log_10_, and SC plus colistin displayed the most significant effect and can ultimately kill all drug‐resistant bacteria during 24 h (Figure 2D).

Relevant studies have shown that the formation of bacterial biofilm can reduce the therapeutic effect of antibiotics.^[^ ^23^ ^]^ In order to explore whether the addition of SFAs can also enhance the effect of colistin on bacterial biofilms, we conducted biofilm formation and eradication experiments. As expected, the low concentrations of drug combinations, which has no direct bactericidal activity, modestly inhibited the biofilm formation of *mcr‐1‐*positive bacteria (Figure S4A, Supporting Information). In addition, in the presence of three SFAs, the eradication effect of colistin on mature biofilm was significantly enhanced (Figure S4B, Supporting Information). Consistently, confocal laser scanning microscope (CLSM) analysis further confirmed the strong elimination ability of SC plus colistin on bacterial biofilms (Figure S4C, Supporting Information). Collectively, the results demonstrate that three SFAs, particularly SC, effectively boost colistin activity against *mcr*‐positive bacteria.

### 2.2 Stability and Safety Evaluation of SFAs and Colistin Combination

The in vivo effect of the drug is influenced by many factors of physiological conditions.^[^ ^24^ ^]^ Therefore, we evaluated the stability of the combination of three SFAs (SCL, SP, and SC) and colistin in the presence of 10% serum and various ions (10 mm), which had almost no impact on the individual MICs of both colistin and SFAs except for Mg^2+^ (Table S1, Supporting Information). We found that after adding Na^+^, K^+^, ethylenediamine tetraacetic acid (EDTA), and serum, the synergistic activity of SFAs and colistin still remained (Table S1, Supporting Information). Specifically, their synergistic activity was enhanced in the EDTA and serum, but reduced or even lost after adding Mg^2+^. The alternation of their synergistic activity caused by EDTA and Mg^2+^ implied that the synergistic mechanisms might be related to the damage of the cell membrane.^[^ ^25^ ^]^

Next, we conducted hemolytic tests on mammalian red blood cells (RBCs) to evaluate the safety of colistin alone or in combination with three SFAs. Consequently, the hemolytic activity of colistin alone or colistin combined with SFAs was lower than 10% (Figure S5A, Supporting Information). In addition, we further evaluated the cytotoxicity of SC alone and the combination of SC and colistin on macrophage RAW264.7 cells. The results showed that when SC was used alone or combined with colistin, the relative cell viability of macrophages was still higher than 90% (Figure S5B, Supporting Information), confirming the safety of SFAs and colistin combinations.

### 2.3 SFAs Suppress the Evolution and Spread of Colistin Resistance

**Figure 3:**
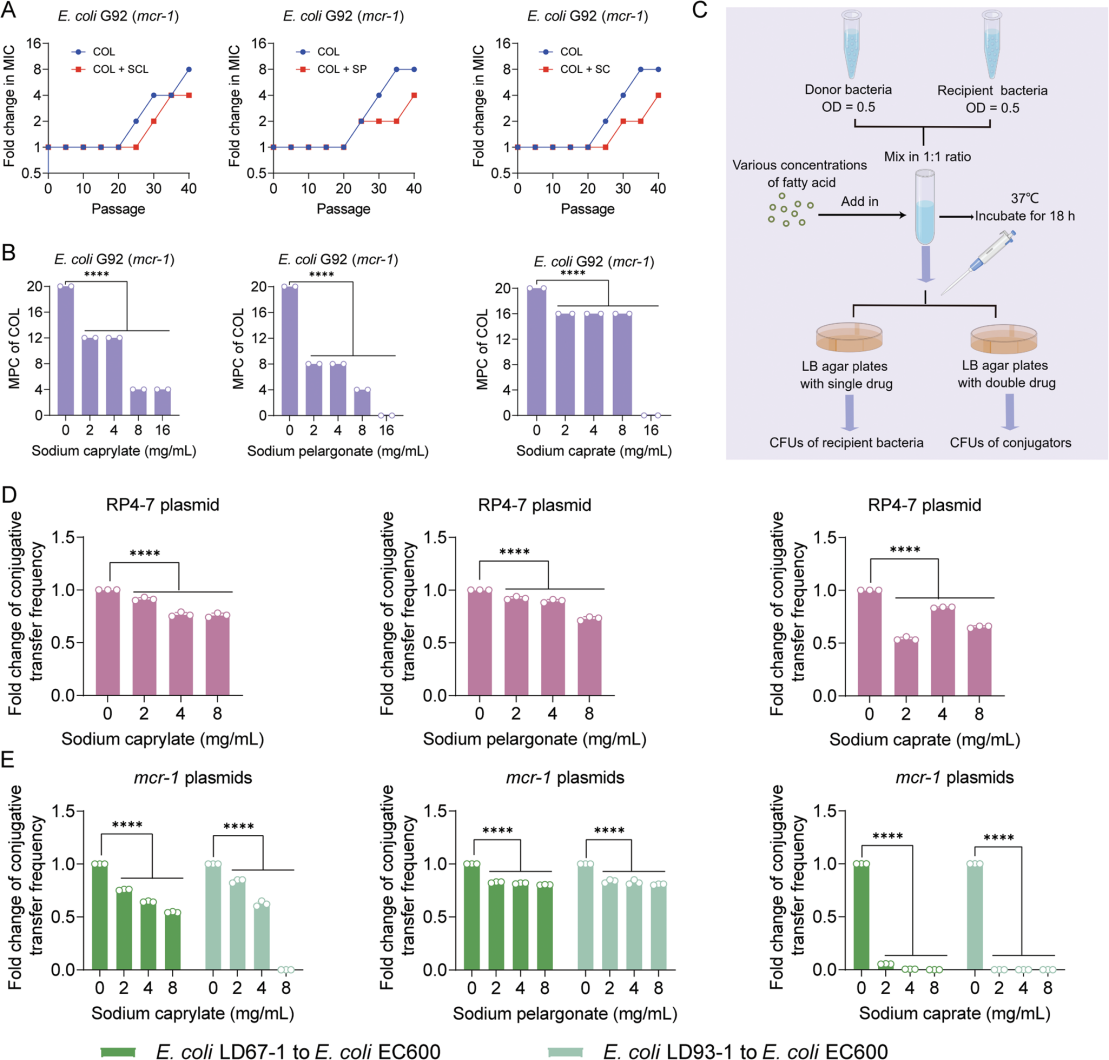
SFAs suppress the evolution of colistin resistance and the transmission of resistance genes. A) Resistance development assays of *E. coli* G92 (*mcr‐1*) after sequential passages with colistin alone or in combination with three SFAs, respectively. B) MPC values of colistin in the existence of increasing concentrations of three SFAs against *E. coli* G92 (*mcr‐1*). C) Schematic illustration of conjugation assays. D,E) Fold changes of conjugative transfer frequency of RP4‐7 plasmid (D) or *mcr‐1*‐carrying plasmids (E) from clinical isolates to the recipient bacteria *E. coli* EC600 in the presence of increasing concentrations of three SFAs. Experiments were conducted with three biological replicates. Data were shown as mean ± SD and one‐way ANOVA was used to evaluate the statistical significance (^****^ *p* < 0.0001).

Bacteria will continue to evolve in the long‐term struggle with antibiotics, resulting in a continued increase in drug resistance. To explore whether three SFAs have the potential to prevent the evolution of colistin resistance, we carried out the resistance development experiment and mutation preventive concentration (MPC) detection. We found that after 40 days of continuous passage, the SFAs (1/8 MIC) modestly slowed down the increase of MICs of colistin (**Figure** **3**A). In addition, the MPC values of colistin against drug‐resistant bacteria were dose‐dependently reduced after exposure to three SFAs (Figure 3B).

Related studies have shown that the *mcr‐1* gene can be widely spread through conjugative plasmids, eventually leading to the dissemination of colistin resistance. Thus, we studied the effect of three SFAs (SCL, SP, and SC) on the conjugative frequency of resistance plasmids using *E. coli* EC600 as the recipient strain (Figure 3C). We found that three SFAs significantly reduced the conjugative transfer frequency of RP4‐7 and *mcr‐1*‐carrying plasmids in a dose‐dependent manner (Figure 3D,E). In particular, SC treatment displayed an excellent inhibitory effect on the conjugation of *mcr‐1*‐harboring plasmids. Together, these results suggest that three SFAs prevent the evolution and spread of mobile colistin resistance.

### 2.4 SFAs Enhance Membrane Permeability and Promote Oxidative Damage

**Figure 4:**
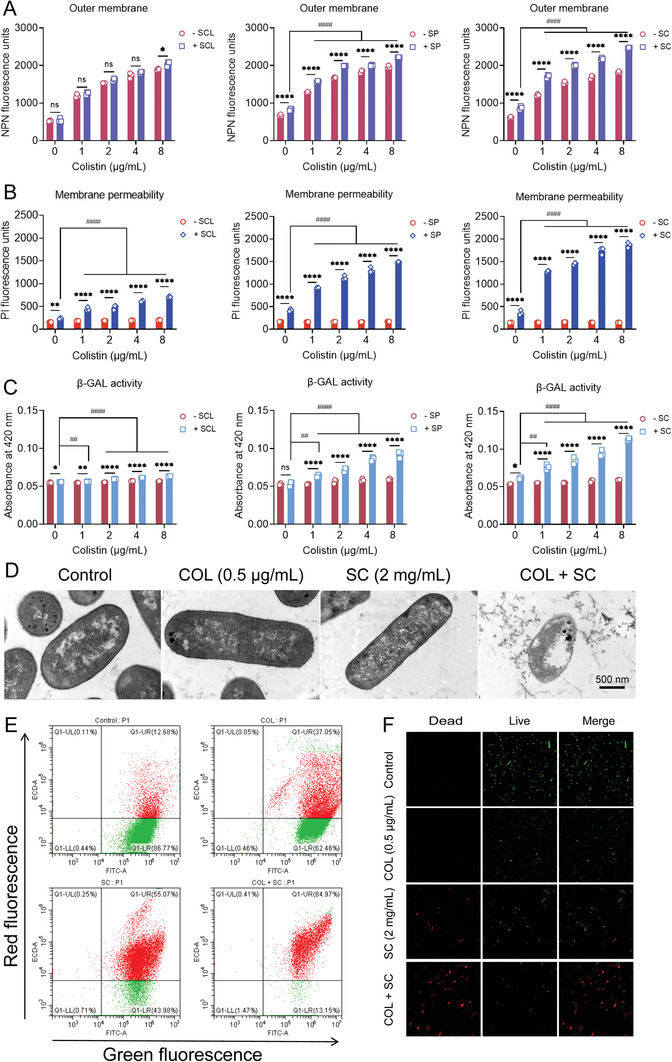
SFAs enhance the damage of colistin to bacterial cell membrane. A,B) Outer membrane (OM) permeability and integral membrane permeability of *E. coli* G92 (*mcr‐1*) in the presence of increasing concentrations of colistin or in combination with SFAs. OM permeability was evaluated by measuring the fluorescence intensity of *N*‐phenyl‐1‐naphthylamine (NPN) after 1 h exposure to colistin alone or in combination with SFAs. Propidium iodide (PI) was used to measure integrity membrane permeability. C) The activity of extracellular β‐galactosidase after exposure to different concentrations of colistin or in combination with SFAs. D) Morphological changes of *E. coli* G92 treated with single or combined drugs visualized with TEM. Scar bar, 0.5 µm. E) The LIVE/DEAD *Bac*Light viability assay of *E. coli* G92 treated with single or combined drugs for 1 h, determined by flow cytometry analysis. Green fluorescence (due to SYTO 9 staining) with an excitation/emission wavelength at 485 nm/498 nm was used to demonstrate viable cells, whereas red fluorescence (due to propidium iodide staining) with an excitation/emission wavelength at 535 nm/615 nm was used to show dead cells. F) Confocal scanning microscope imaging results of the living and dead status of *E. coli* G92 when SFAs or colistin was added alone or in combination. Dead bacteria were labeled red and living bacteria were dyed green. Experiments were carried out with three biological replicates and data were given as mean ± SD. Two‐way ANOVA was used to evaluate the statistical significance between colistin plus SFAs and colistin (ns, not significant, ^*^ *p* < 0.05, ^**^ *p* < 0.01, ^****^ *p* < 0.0001). Statistical significance of colistin plus SFAs versus SFAs was determined by one‐way ANOVA, and shown as ^##^ *p* < 0.01, ^####^ *p* < 0.0001.

Colistin kills Gram‐negative bacteria through specific interaction with LPS of the bacterial outer membrane. Also, colistin resistance is mainly mediated by LPS modification and the decreased affinity between colistin and LPS. Thus, we hypothesized whether the addition of SFAs could enhance the damage of colistin on bacterial cell membrane compared with colistin alone. First, we used a hydrophobic fluorescent probe *N*‐phenyl‐1‐naphthylamine (NPN) to evaluate the effect of three SFAs on the outer membrane permeability of *E. coli* G92. Compared with the effect of colistin alone, the addition of 1 mg mL^−1^ SP or SC resulted in enhanced outer membrane permeability, but the effect of SCL was weak (**Figure** **4**A). In addition, the results of the propidium iodide (PI) assay showed that the combination of colistin and SFAs led to the enhancement of membrane permeability compared with colistin or SFAs alone (Figure 4B). In contrast, colistin alone displayed a weak effect on the fluorescence units, which maybe because MCR‐1 protects the cytoplasmic membrane from damage caused by colistin.^[^ ^26^ ^]^ Consistently, after adding 1 mg mL^−1^ SFAs, the activity of β‐galactosidase in *E. coli* G92 exposed to colistin was enhanced compared with colistin or SFAs alone (Figure 4C), due to the release of β‐galactosidases from cells after membrane rupture. In addition, we also found that SFAs at sub‐inhibitory concentrations can also cause membrane damage in a dose‐dependent manner, but the damage effect was much weaker than the combined effect (Figure S6, Supporting Information). Next, transmission electron microscope (TEM) analysis was applied to monitor membrane damage induced by the combination of SC and colistin. Consequently, bacterial cells after exposure to one‐quarter MIC of SC or colistin showed complete cell morphology and membrane structure, whereas bacterial cell membrane was obviously damaged in the combined drug group, accompanied by the leakage of cell contents (Figure 4D). Live/dead cells staining was performed using fluorescent dyes SYTO 9 and propidium iodide (PI) via flow cytometry and confocal scanning microscope analysis. Consistently, the results showed that the proportion of dead cells (red fluorescence) was significantly increased after the combined treatment of SC and colistin for 1 h compared with the monotreatment (Figure 4E,F). These findings reveal that SFAs drastically enhance the damage of colistin to drug‐resistant bacterial membrane.

**Figure 5:**
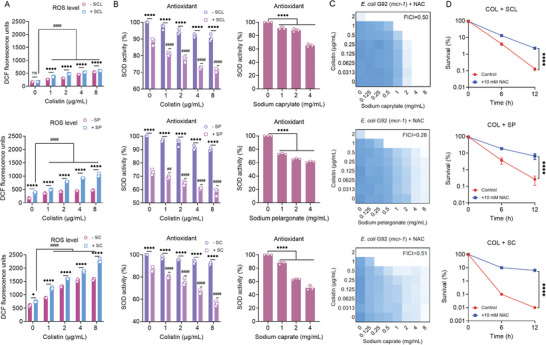
Aggravated oxidative damage is essential for the synergism of SFAs and colistin. A) ROS levels of *E. coli* G92 (*mcr‐1*) treated by increasing concentrations of colistin or in combination with three SFAs (1 mg mL^−1^). A fluorescence probe 2′,7′‐dichlorodihydrofluorescein diacetate (DCFH‐DA) was used to monitor the levels of ROS in cells (*λ*excitation = 488 and *λ*emission = 525 nm). B) SOD activity assay of *E. coli* G92 (*mcr‐1*) after exposure to colistin or three SFAs alone or their combination. C,D) The addition of ROS scavenger NAC abolishes the potentiation of three SFAs to colistin against *E. coli* G92 (*mcr‐1*) through checkerboard assay (C) and time‐killing curves (D). Experiments were carried out with three biological replicates and all data were given as mean ± SD, and one‐way/two‐way ANOVA was used to determine the statistical significance (ns, not significant, ^*^ *p* < 0.05, ^****^ *p* < 0.0001). Statistical significance of colistin plus SFAs versus SFAs was determined by one‐way ANOVA, and shown as ^##^ *p* < 0.01, ^####^ *p* < 0.0001.

Membrane damage is generally associated with the production of reactive oxygen species (ROS), which plays a vital role in the antibiotic‐mediated killing of bacteria. We further analyzed the production of ROS in bacteria after exposure to three SFAs (SCL, SP, and SC). As expected, the combination of SFAs and colistin significantly promoted the intracellular accumulation of ROS in cells compared with the colistin or SFAs alone (**Figure** **5**A). Superoxide dismutases (SOD) are a group of enzymes that catalyze the dismutation of O^2−^ to O_2_, providing cellular defense against ROS. Also, we found that compared to colistin alone, the addition of SFAs resulted in a dose‐dependent decrease in SOD activity (Figure 5B). Consistently, the addition of ROS scavenger *N*‐acetyl‐L‐cysteine (NAC) reduced their synergistic bacteriostatic and bactericidal activity, respectively (Figure 5C,D), indicating that the generation of ROS is indispensable for its combined antibacterial activity.

### 2.5 SFAs Impair Proton Motive Force‐Dependent Efflux Pump

Relevant studies have shown that the insertion of specific membrane destruction compounds into lipid bilayer usually leads to great changes in membrane fluidity, thereby resulting in the loss of membrane proteins, leakage of cellular compounds, and bacterial death.^[^ ^27^ ^]^ A membrane‐sensitive dye Laurdan was used to detect the effect of three SFAs membrane fluidity of *E. coli* G92. The results showed that the addition of sub‐inhibitory concentrations of SFAs induced decreased fluorescence intensity, indicating that the membrane fluidity was increased (Figure S7, Supporting Information). The change in membrane stiffness will affect the internal stability of bacteria, leading to primary metabolic disorders, such as the dissipation of proton motive force (PMF).

**Figure 6:**
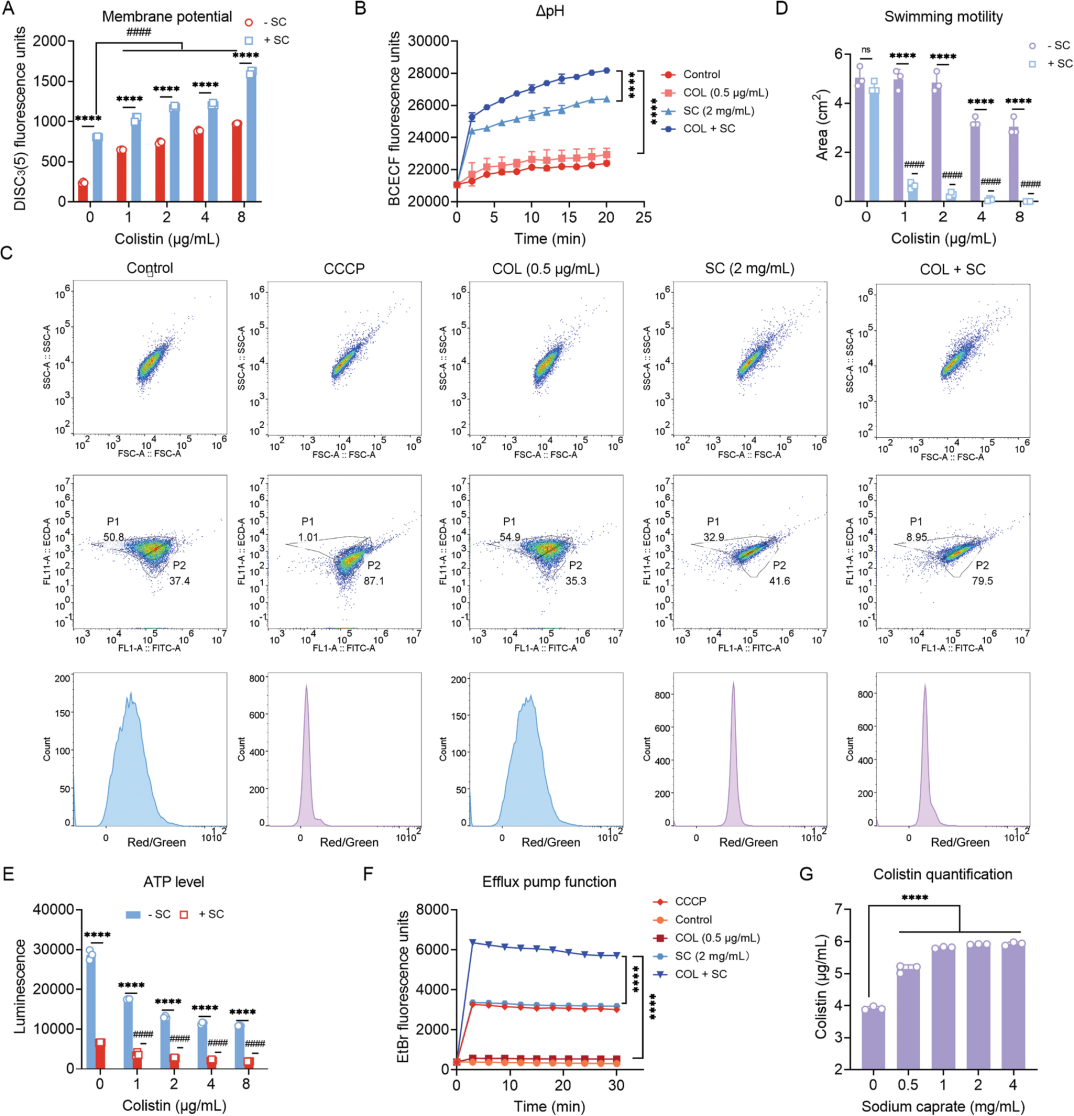
SC impairs bacterial proton motive force‐dependent efflux pump function. A) Membrane potential of *E. coli* G92 under colistin alone or in combination with SC. Fluorescence intensity of DiSC_3_(5) in *E. coli* G92 after treatment with colistin alone or in combination with SC was monitored. B) Transmembrane proton gradient (ΔpH) in BCECF‐AM‐labeled bacterial cells under the colistin, SC alone, or their combination. C) Proton motive force (PMF) measurement by flow cytometry analysis. CCCP was used as the positive control. D) Swimming motility assay of *E. coli* G92 after treatment with colistin alone or in combination with SC. Overnight cultures were adjusted to OD_600_ of 0.5, and inoculated on 0.3% agar plates for 48 h at 37°C. E) Intracellular ATP levels of *E. coli* G92 exposed to colistin alone or in combination with SC, measured by monitoring the corresponding luminescence signals. F) Bacterial efflux pumps activity in *E. coli* G92 after treatment with the colistin, SC alone, or their combination, determined using the EtBr (*λ*excitation = 530 and *λ*emission = 600 nm). G) Intracellular colistin accumulation assay of *E. coli* G92 after treatment with different concentrations of SC. Experiments were carried out with three biological replicates and data were given as mean ± SD, and one‐way/two‐way ANOVA was used to determine statistical significance (ns, not significant, ^****^ *p* < 0.0001). Statistical significance of colistin plus SFAs versus SFAs was determined by one‐way ANOVA, and shown as ^####^ *p* < 0.0001.

PMF is essential for bacterial survival and consists of both the membrane potential (ΔΨ) and the transmembrane proton gradient (ΔpH).^[^ ^28^ ^]^ First, we used a fluorescent dye 3,3′‐dipropylthiadicarbocyanine iodide (DiSC_3_(5)) to detect the effect of SFAs on membrane potential. The fluorescence value of this dye increased when ΔΨ was disrupted, and our results showed that SC plus colistin led to an increase in fluorescence values compared to colistin alone, suggesting the dissipated ΔΨ (**Figure** **6**A), while a weaker effect was observed in the presence of SCL and SP (Figure S8A, Supporting Information). The disruption of ΔΨ would be compensated by increasing ΔpH, thus we evaluated the effect of the combination on ΔpH using a fluorescent probe 2′,7′‐bis‐(2‐carboxyethyl)−5‐(and‐6)‐carboxyfluorescein, acetoxymethyl ester (BCECF‐AM). Interestingly, we found that the fluorescence intensity increased after the combination treatment of SC/SP and colistin, indicating the upregulation of ΔpH, but no for SCL (Figure 6B; Figure S8B, Supporting Information). Furthermore, we re‐examined PMF changes using a fluorescence probe 3,3′‐diethyl‐oxo‐iodocarbocyanine (DiOC_2_(3)) by flow cytometry. In agreement with the above results, the drug combination led to the destruction of PMF (Figure 6C). We next analyzed the final effect of the combination of drugs on PMF by swimming motility test. As shown in Figure 6D, compared with colistin alone, the addition of SC significantly inhibited bacterial swimming motility, with a decreasing trend in motility area. Similar results were also observed for SCL and SP (Figures S8C and S9, Supporting Information), further confirming the interrupted PMF. Meanwhile, the results also indicated that the inhibitory effects of colistin or SFAs alone on motility were not as strong as the combined effect (Figures S8D and S9, Supporting Information). Given that the destruction of PMF would affect the synthesis of adenosine 5′‐triphosphate (ATP), we tested the ATP content and the results showed that the intracellular ATP levels were reduced under SC/SP plus colistin (Figure 6E; Figure S10A, Supporting Information). Considering that bacterial efflux pump is highly dependent on PMF‐driven ATP synthesis, we next applied ethidium bromide (EtBr) dye to investigate the impact of three SFAs on the efflux pump, which mediates drug resistance. Consistently, the results showed that the function of bacterial efflux pumps was remarkably inhibited after exposure to SC or SP, similar to the effect of efflux pump inhibitor carbonyl cyanide 3‐chlorophenylhydrazone (CCCP) (Figure 6F; Figure S10B, Supporting Information). Finally, we monitored the intracellular accumulation of colistin in bacteria under SC treatment by enzyme‐linked immunosorbent assay (ELISA) assay and found that SC led to increased colistin contents in cells (Figure 6G). Together, our findings indicate that three SFAs, particularly SC, impair the function of bacterial efflux pumps by dissipating PMF, thereby enhancing the intracellular accumulation of colistin and achieving boosted antibacterial activity.

### 2.6 SC Reduces the Modification of LPS and Inhibits MCR‐1 Protein

**Figure 7:**
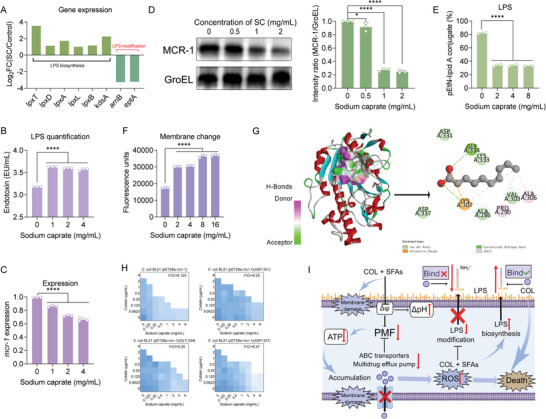
Molecular mechanisms underlying the synergistic activity of SC and colistin. A) mRNA expression of LPS biosynthesis and LPS modification‐related genes through transcriptome analysis. B) LPS quantitative analysis of *E. coli* G92 treated by SC. C) mRNA expression of colistin resistance gene *mcr‐1* in the presence of increasing concentrations of SC. D) Western blot assays of MCR‐1 protein after exposure to SC. E) SC decreases the modification of lipid A by pEtN through MCR‐1 protein. The percentage of pEtN‐lipid A conjugate was determined based on LC‐MS analysis. F) Change of positive charge on the membrane surface of *E. coli* G92 after treatment with SC. G) Molecular docking analysis of SC and MCR‐1 protein. The interactions and binding sites in MCR‐1 were shown using a 2D diagram. H) Synergistic activity of SFAs with colistin against engineered *E. coli* BL21 (pET28a‐*mcr‐1*) and its MCR mutants. I) Schematic illustration the synergistic mechanisms of colistin and SC. The combination treatment of colistin and SFAs enhances membrane damage and dissipates PMF‐dependent efflux pump, thus resulting in intracellular colistin accumulation and ROS generation. Crucially, SFAs promote LPS biosynthesis and inhibit the modification of LPS, thereby restoring the affinity between colistin and drug‐resistant bacteria. Experiments were carried out with three biological replicates and all data were given as mean ± SD, and one‐way ANOVA was used to determine statistical significance (^*^ *p* < 0.05, ^****^ *p* < 0.0001).

Having shown the mechanisms by which SFAs enhance the antibacterial activity of colistin against *mcr*‐positive bacteria, we next sought to elucidate how SFAs initiate these actions using SC as an example owing to its strongest potentiation. To this end, we performed the transcriptional analysis of *E. coli* (*mcr‐1*) after 4 h of SC treatment. Consequently, SC exposure led to 1000 upregulated differentially expressed genes (DEGs) and 981 downregulated DEGs (Figure S11A, Supporting Information). Gene Ontology (Go) and Kyoto encyclopedia of genes and genomes (KEGG) enrichment analysis revealed that DEGs with increased expression were involved in ribosome and DEGs with repressed expression were involved in quorum sensing and biofilm formation (Figure S11B,C, Supporting Information). Specifically, we found the upregulation of LPS biosynthesis and 30S/50S ribosome‐related genes, the latter possibly due to negative feedback regulation caused by protein synthesis inhibition. However, two‐component system, ATP‐binding cassette (ABC) transporter, antioxidant function, gamma‐aminobutyric acid (GABA) shunt, LPS modification, multidrug efflux pump, and quorum sensing‐related gene expression were significantly suppressed (**Figure** **7**A; Figure S11D, Supporting Information). Notably, increased LPS biosynthesis and decreased LPS modification favored the antimicrobial effect of colistin, and the multidrug efflux pump encoded by the *mdt* gene was associated with colistin resistance in Gram‐negative bacteria.^[^ ^29^ ^]^ Further real‐time quantitative PCR (RT‐qPCR) analysis of representative gene expression profiles was consistent with the transcriptional results (Figure S12, Supporting Information). The above results inspire the action of SC may be related to increased LPS synthesis and decreased LPS modification. To verify this, we conducted LPS quantitative detection in *E. coli* G92 (*mcr‐1*) after exposure to SC. In agreement with the RNA sequencing, SC significantly promoted the production of LPS (Figure 7B). Furthermore, we determined the expression of MCR‐1 under SC treatment by RT‐qPCR and Western blot analysis. Intriguingly, SC drastically reduced the transcription and translation of *mcr‐1* gene (Figure 7C,D). To investigate whether the upregulation of LPS synthesis and downregulation of LPS modification‐related genes could eventually prevent the modification of lipid A by pEtN, the proportion of modified lipid A in *E. coli* G92 under different concentrations of SC was determined by liquid chromatography‐mass spectrometry (LC‐MS). As expected, pEtN‐lipid A conjugate was significantly reduced in *E. coli* G92 (*mcr‐1*) after the addition of SC (Figure 7E). We further tested whether these changes would eventually affect bacterial membrane charge using FITC‐labeled poly‐L‐lysine (PLL). The results showed the increased fluorescence value under SC, suggesting a decreased positive charge on the bacterial membrane surface (Figure 7F). These data support the enhanced action of colistin on bacterial membrane in the presence of SFAs. In addition to the inhibitory effect of SC on MCR expression, we further explored whether SC could directly bind to MCR protein using molecular docking. Interestingly, docking analysis using MCR‐1 as a receptor and SC as a ligand showed that SC had a high affinity for MCR‐1 with a binding energy of −4.2 kcal mol^−1^. Specifically, SC can attach to MCR‐1 protein through Van del Waals (Ala286, Val303, Asp331, Lys333, and Asp337), hydrogen bond with Gly334, and attractive charge with Lys307 (Figure 7G). To verify these binding sites, we constructed mutants in the engineered *E. coli* BL21 (pET28a‐*mcr‐1*) and found mutants at Asp331, Gly334, and Asp337 displayed decreased synergistic activity, suggesting the crucial roles of these sites in the SC‐MCR interaction (Figure 7H). Collectively, these mechanistic studies demonstrate that SFAs enhance colistin activity by promoting oxidative damage and LPS biosynthesis, preventing LPS modification, and inhibiting the efflux pump function (Figure 7I).

### 2.7 Restoration of Therapeutic Efficacy of Colistin by SC In Vivo

**Figure 8:**
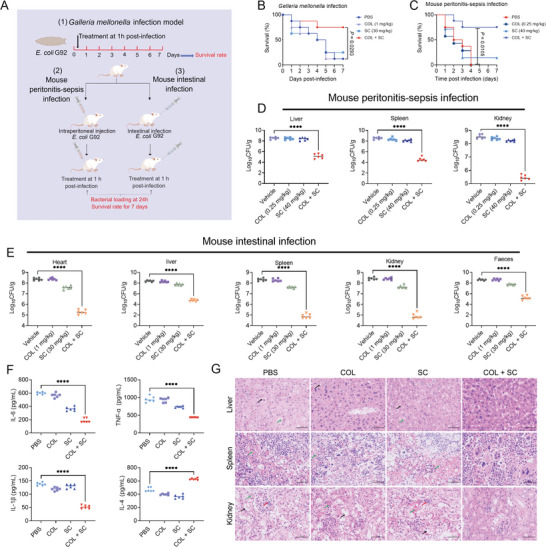
SC rescues colistin activity in three animal models of infection. A) Schematic illustration of experimental protocols for three animal models. B) Survival rates of the *G. mellonella* larvae (n = 8 per group) infected by colistin‐resistant *E. coli* G92 and treated with colistin (1 mg kg^−1^) and SC (30 mg kg^−1^) alone or their combination. *p* values were determined by log‐rank (Mantel–Cox) test. C) Survival rate of mice infected by colistin‐resistant *E. coli* G92 for 7 days post‐infection. ICR mice (n = 8 per group) were given a lethal dose of *E. coli* G92 (3 × 10^8^ CFUs), and treated with a single dose of colistin (0.25 mg kg^−1^), SC (40 mg kg^−1^), a combination of colistin plus SC (0.25 + 40 mg kg^−1^), or PBS by intraperitoneal injection. *p* values were determined by log‐rank (Mantel–Cox) test. D) Bacterial loads in the mice organs (liver, spleen, and kidney) in the mice peritonitis‐sepsis infection model. *p* values were determined by Mann–Whitney U test. Lines indicate the median for each group. E) In intestinal infection model, bacterial suspension (5.0 × 10^8^ CFUs) was gavage into the female ICR mice (n = 6 per group) and treated with colistin (1 mg kg^−1^), SC (30 mg kg^−1^), or their combination at 1 h after infection. After 24 h post‐infection, bacterial loads in mice heart, liver, spleen and kidney, and feces were determined. *p* values were determined by Mann–Whitney U test. Lines indicate the median for each group. F) Statistical analysis of inflammatory cells after different treatments. Levels of IL‐6, TNF‐α, IL‐1β, and IL‐4 in liver tissue. Experiments were carried out with six biological replicates and lines indicate the median for each group, and one‐way ANOVA was used to determine statistical significance (^****^ *p* < 0.0001). G) Histopathological observation of liver, spleen, and kidney after different treatments. Arrows in liver tissue show cytolysis (black) and vacuoles (green). The arrow in the spleen shows congestion (green). The arrow in the kidney tissue shows the swelling of renal tubular epithelial cells (black), inflammatory cell infiltration (red), and hyperemia (green).

Given that the combination of colistin and SC exhibited excellent synergistic bactericidal activity against resistant bacteria in vitro, we next explored whether SC could reverse *mcr*‐mediated colistin resistance in vivo, thereby restoring its clinical efficacy. To this end, we tested the in vivo efficacy of their combination in three animal models of infection (**Figure** **8**A). In the *Galleria mellonella* infection model, the survival rate of infected larvae treated with PBS or colistin was only 12.5% within seven days of infection. By contrast, combination therapy achieved 75% survival, which was significantly higher than monotherapy (Figure 8B). Similarly, in the mouse peritonitis‐sepsis infection, the combination of SC and colistin resulted in 75% survival, while mice all died in colistin monotreatment (Figure 8C). Consistently, > 2log_10_ reduction of bacterial loads in liver, spleen, and kidney under combination therapy was observed (Figure 8D). Furthermore, we assessed the efficacy of colistin and SC (1 + 30 mg kg^−1^) in a mouse model of intestinal infection. Intriguingly, bacterial loads in four organs (heart, liver, spleen, and kidney) and feces were significantly reduced under combination treatment (Figure 8E).

Next, we evaluate the effect of different treatments on inflammation using an ELISA assay. In contrast to the control group, we found that the levels of pro‐inflammatory cytokines such as tumor necrosis factor‐α (TNF‐α), interleukin‐6 (IL‐6), and IL‐1β were much lower in liver samples from mice treated with SC in combination with colistin, while the levels of anti‐inflammatory cytokine IL‐4 was increased under combination group (Figure 8F). Moreover, the results of histopathological analysis showed that there were cell lysis and vacuoles in the liver, congestion in the spleen, swelling of renal tubular epithelium, hemorrhage, and inflammatory cell infiltration in the kidney of control and colistin alone groups (Figure 8G). By contrast, in the combined treatment group, there were no noticeable pathological changes in all tissues. These data confirm that SC effectively rescues the activity of colistin in vivo and inhibits inflammatory response.

## 3 Discussion

The increasing prevalence of MDR bacteria is a serious threat to public health. Colistin is a clinically important antibiotic for the treatment of infections caused by MDR Gram‐negative bacteria. However, the dissemination of novel colistin resistance gene *mcr* has dramatically challenged the clinical application of colistin, it is urgent to develop new antimicrobial strategies such as colistin enhancers. A variety of fatty acids exist in the diet, bloodstream, cells and tissues of humans, which possess various biological activities, such as serving as energy sources and membrane constituents. The structure‐activity relationship analysis indicates that the fatty acyl chain plays an indispensable role in the antibacterial activity of polymyxins. This evidence inspires us to explore the potentiation of SFAs to colistin in the fight against drug‐resistant bacteria. In this study, we found that SFAs with a weak direct antibacterial effect drastically potentiated colistin activity against *mcr*‐carrying bacteria, among which SC and colistin had the most potent synergistic antibacterial effect both in vitro and in vivo. Notably, SC is approved as a safe food additive in both the United States (US) and the European Union (EU), and there are no daily intake restrictions.^[^ ^30^ ^]^

It is well known that one of the limiting factors for the clinical application of colistin is its strong nephrotoxicity and neurotoxicity in mammals.^[^ ^31^ ^]^ Therefore, the application of low‐dose colistin and the development of colistin antidotes are crucial for its clinical application. For example, minocycline was found to reduce colistin‐induced neurotoxicity by inhibiting oxidative stress and mitochondrial dysfunction.^[^ ^32^ ^]^ Importantly, in this study, we found that the combined use of SC and colistin can remarkably reduce the dosage of colistin to 0.25 mg kg^−1^, which is > 10 times lower than the clinically recommended dosages of colistin (2.5–5 mg kg^−1^ per day divided every 12 h). In addition, SC has been clinically used to enhance the rectal absorption of the low‐molecular‐weight drug ampicillin^[^ ^33^ ^]^ and has been widely proven to improve the bioavailability of poorly absorbed drugs.^[^ ^34^ ^]^ Therefore, we reasoned that the use of SC as a potentiator might promote the intestinal absorption of colistin, which would strongly expand its clinical values and improve its effectiveness. However, the beneficial role of SC in the intestinal absorption of colistin remains to be explored.

Colistin is a positively charged cyclic lipopeptide antibiotic that disrupts bacterial cell membrane via electrostatic interaction with LPS located on the outer membrane, while the chemical modification of lipid A by enzymes, including MCR or phosphoethanolamine transferase (EptA), reduces this interaction. Thus, membrane damage and resulting oxidative damage are important indicators for the antibacterial activity of colistin. For example, our previous study indicated that melatonin potentiated colistin activity by enhancing bacterial outer membrane permeability and promoting oxidative damage.^[^ ^18^ ^]^ Consistently, in this study, we also found that the combined use of SFAs and colistin resulted in enhanced membrane permeability and oxidative damage of *mcr*‐positive bacteria compared with colistin alone. Moreover, we revealed that SC impaired the functions of multidrug efflux pumps by dissipating bacterial PMF, which is essential for ATP synthesis via the F1F0‐ATPase and the transport of various substances.^[^ ^35^ ^]^ The decreased drug efflux further resulted in enhanced intracellular accumulation of colistin in *E. coli*, which is necessary for antibiotics to kill Gram‐negative bacteria.^[^ ^36^ ^]^

Furthermore, we clarified the most fundamental cause that mediates the potentiation of SC to colistin. Interestingly, we found that the addition of SC led to an increase in LPS biosynthesis, and at the same time, the expression levels of LPS modification‐related enzymes, particularly MCR‐1, were significantly reduced under SC treatment. Consequently, the proportion of modified LPS with pEtN was decreased, resulting in enhanced interaction between colistin and bacterial membrane. Meanwhile, we also demonstrated that SC can directly bind to the active center of MCR‐1 (amino acid residue 330–350).^[^ ^37^ ^]^ Consistently, the mutations in these binding sites reduced the synergism between SC and colistin.

## 4 Conclusion 

In conclusion, our study shows that three SFAs, particularly SC, effectively resensitize *mcr*‐positive bacteria to colistin both in vitro and in vivo. Mechanistic studies indicate that SFAs reduce LPS modification by simultaneously promoting LPS synthesis and inhibiting the activity of MCR protein, enhance membrane damage and impair PMF‐dependent efflux pump, thereby enhancing colistin activity. The discovery of SFAs as novel colistin enhancers provides a promising therapeutic strategy to address the increasingly serious threat of colistin‐resistant Gram‐negative bacterial infections.

## 5 Experimental Section

### Bacterial Strains and Reagents

In Table S2 (Supporting Information), all strains utilized in this study were mentioned. Bacteria were kept in the nutritional broth with 20% (v/v) glycerol at −80°C. All strains were cultivated in Mueller–Hinton Broth (MHB) or on LB agar (LBA) plates for the following tests. Chemical reagents were procured from Aladdin (Shanghai, China) or Beyotime (Shanghai, China), while antibiotics were purchased from the China Institute of Veterinary Drug Control.

### MIC Determinations

The MICs of drugs were determined using the micro‐broth dilution method with reference to CLSI 2021.^[^ ^38^ ^]^ Briefly, bacteria reaching the exponential phase were diluted to MHB in a 1:1000 ratio. Then the bacterial suspension (1.5 × 10^6^ CFUs per mL) and drugs of different concentrations were mixed in equal volumes in a sterile 96‐well microtiter plate. After 18 h of incubation at 37°C, MIC values were defined as the lowest drug concentration at which there was no visible bacterial growth.

### Checkerboard Assays

Checkerboard tests were used to assess the synergistic activity of antibiotics and SFAs. In brief, 100 µL of MHB was added into each well of a 96‐well plate with an 8 × 8 matrix. The antibiotics and SFAs were then diluted twice along the abscissa and ordinate, respectively. After incubating at 37°C for 18 h with bacterial suspension (1.5 × 10^6^ CFUs per mL), the absorbance of each well at 600 nm was measured. The mean absorbance at 600 nm of biological replicates was presented for the following the FIC index (FICI). The formula is as follows: FIC index = FICIa + FICIb = MICab/MICa + MICba/MICb. Synergy was indicated by the FIC index of ≤ 0.5.^[^ ^39^ ^]^

Checkerboard tests were carried out to assess the impact of metal ions, EDTA, and serum on the synergistic activity between SFAs and colistin. In this treatment, 10% serum and 10 mm of Na^+^, K^+^, Mg^2+^, or EDTA were added into the MHB. In addition, ROS scavenger *N*‐acetyl‐cysteine (NAC, 10 mm) was added to MHB and thoroughly mixed before the checkerboard assays.

### Safety Assessment

The hemolysis of colistin alone and in combination with SFAs was evaluated.^[^ ^40^ ^]^ Briefly, sheep blood cells at 8% of volume were treated at 37°C for 1 h with 0–8 µg mL^−1^ of colistin alone or in combination with 2 mg mL^−1^ of SFAs. Triton X‐100 (0.2%) and phosphate buffer saline (PBS) were employed as a positive and blank control, respectively. Using an Infinite E Plex Microplate reader (Tecan), the absorbance of the released hemoglobin was measured at 576 nm. Finally, the hemolysis rate (%) was calculated accordingly.

Macrophage RAW264.7 was cultured in a 37°C sterile incubator containing 5% CO_2_, and was used for cytotoxicity studies when the density of 5000 cells per well in a 96‐well microtiter plate. After 36 h, various concentrations of SC or combinations of SC (1 mg mL^−1^) and colistin were added to the culture medium. The culture medium without added cells and the cells without added drugs were used as the control group and blank group, respectively. A CCK8 kit was used to assess cell viability following a 24 h incubation period. Wells received 10 µL CCK8 regent, and cells were then allowed to incubate for 30 min. The optical density at the wavelength of 450 nm was monitored. The following equation was used to calculate the cell viability.

(1) $$\begin{array}{ccc} \mathrm{Cell}\;\;\mathrm{viability} & = & \left({\mathrm{OD}}_{\mathrm{sample}}-{\mathrm{OD}}_{\mathrm{blank}}\right)/\left({\mathrm{OD}}_{\mathrm{control}}-{\mathrm{OD}}_{\mathrm{blank}}\right)\\ & & \times 100\% \end{array}$$

### Time‐Dependent Killing Curves

Bacteria were diluted into MHB at 1:1000. Then, the bacteria were treated with SFAs (2 mg mL^−1^) and colistin (0.5 µg mL^−1^) alone or in combination, while the control group was not treated with drugs. Samples were serially diluted 10‐fold and plated on MHA plates at each time point (0, 6, 12, and 24 h). The number of primary CFUs per mL was calculated after overnight incubation of bacterial colonies.^[^ ^41^ ^]^

### Biofilm Formation Determination

Colistin (final concentration from 0 to 1 µg mL^−1^) was added into *E. coli* G92 suspension (1 × 10^7^ CFU mL^−1^) in the presence or absence of 2 mg mL^−1^ SFAs. Bacteria in the absence of any drug was used as a negative control. Bacteria were incubated at 37°C for 36 h under static conditions. Cells were washed three times with 300 µL PBS. Next, 200 µL methanol was added to fix the cells for 15 min, and 0.1% crystal violet was added to the stain for 15 min. After the dye solution was eliminated, the stained biofilm was washed three times with PBS and then dried naturally. Finally, 33% glacial acetic acid (100 µL) was used to dissolve the crystal violet‐stained biofilm, which was then incubated at 37°C for 30 min. The mass of biofilms was calculated by measuring the absorbance of the supernatant at 570 nm.^[^ ^24^ ^]^

### Biofilm Eradication Assay

Overnight *E. coli* G92 was diluted 1/100 in MHB, and the mixture was incubated at 37°C with shaking at 200 rpm for 6 h. Then, in a 96‐well microtiter plate, 100 µL of bacterial suspensions were combined with an equal volume of MHB. The planktonic bacteria were eliminated over a 36 h at 37°C incubation period. Then, biofilm cells were either treated with 2 mg mL^−1^ of SFAs alone or in conjunction with 4–16 µg mL^−1^ colistin. The remaining cells were spread out using the ultrasonic treatment for 20 min after 2 h of incubation at 37°C. After being redissolved in PBS and 10 times diluted, the mixture was then overnight plated on LB agar plates at 37°C. After 18 h incubation, the colonies were counted accordingly.

### Resistance Development Study

*E. coli* G92 overnight cultures were diluted 1:100 into LB broth containing 1/8 × MIC of colistin or combined with 1/8 × MIC of SFAs. The bacterial culture was diluted 1:100 and transferred to a fresh medicated medium after 12 h of incubation at 37°C to start the subsequent generation. MIC values were determined after every five passages. The serial passage was continued for 40 days.

### Mutation Preventive Concentration (MPC) Assay

In LB agar plates, colistin or its combination with the SFAs was added in varying concentrations. *E. coli* G92 (100 µL) at a concentration of 1.0 × 10^10^ CFU were plated onto the matching resistant agar plates, and the plates were then incubated at 37°C. After 72 h, bacterial growth was observed, and the drug's MPC was determined to be the lowest concentration that could stop resistance development (mutant colonies).^[^ ^42^ ^]^

### Conjugation Assays

Conjugation experiments were carried out by monitoring the conjugative transfer frequency between the donor and recipient in the presence or absence of SFAs.^[^ ^43^ ^]^ *E. coli* DH5α containing RP4‐7 plasmid, *E. coli* LD93‐1, and *E. coli* LD67‐1 containing *mcr‐1*‐positive plasmid were used as donor bacteria, respectively. The recipient bacteria, *E. coli* EC600, carried the gene for rifampicin resistance. The bacteria were cultured at 37°C and then collected by centrifugation and resuspended in LB broth to an OD_600_ of 0.5. The donor and recipient were combined in a volume of 2 mL in a 1:1 ratio, and then different concentrations of the SFAs (0–8 mg mL^−1^) were added to the mixture. After an 18 h incubation period at 37°C, the mixture was serially diluted and plated on LB agar plates with single or double antibiotics. Calculations of conjugators and conjugation frequencies were performed using bacterial CFU counts.

### Outer Membrane and Cell Membrane Permeability

The fluorescent dyes *N*‐phenyl‐1‐naphthylamine (NPN) and propidium iodide (PI) were used to evaluate the permeability of the bacterial outer membrane and cell membrane, respectively.^[^ ^44^ ^]^ Briefly, bacterial suspensions were incubated with final doses of NPN (10 µM) and PI (5 µm) for 30 min, and then colistin alone (0–8 µg mL^−1^) or in combination with SFAs (1 mg mL^−1^) was applied for 1 h. Using an Infinite E Plex Microplate reader (Tecan) with excitation/emission wavelengths of 350 nm/420 nm (NPN) and 535 nm/615 nm (PI), the permeability of the outer/cell membrane was then measured.

### β‐Galactosidase Activity Assay

The *E coli* G92 cultured overnight was expanded in a 1/100 ratio for 4 h. The bacterial cultures were centrifuged at 4500 g for 5 min, then resuspended with PBS to adjust an OD_600_ of 0.5, and then split into new 2 mL tubes. Bacterial suspension was treated for 4 h with colistin (0–8 µg mL^−1^), or a combination of colistin and SFAs (1 mg mL^−1^). After another centrifugation, take 200 µL supernatant onto a 96‐well microtiter plate. Finally, O‐nitrophenyl‐D‐galactopyranoside (ONPG) with a final concentration of 3 mm was added to each well and incubated at 37°C for 1 h. The absorbance at 420 nm was measured.

### Transmission Electron Microscopy (TEM) Assay

Briefly, overnight cultured bacteria suspensions were cleaned twice and were treated by SC (2 mg mL^−1^) and colistin (0.5 µg mL^−1^) alone or their combination, with no treatment was applied as control group, for 1 h at 37°C. After incubation, the cell pellets were extracted, twice‐washed in PBS, and fixed with 2.5% glutaraldehyde overnight at 4°C. Subsequently, the morphological and intracellular alterations of bacterial cells were investigated using TEM.

### Membrane Fluidity Assay

After centrifuging and washing with PBS, the exponential bacterial suspensions were incubated at 37°C for 30 min in the dark with 10 µm Laurdan. The labeled cell culture was washed twice with PBS and incubated with different amounts of SFAs at 37°C for 1 h. Using an Infinite E Plex Microplate reader (Tecan) with emission wavelengths of 435 and 490 nm upon excitation at 350 nm, the Laurdan fluorescence levels were determined. The Laurdan GP was calculated using the formula GP = (I435 − I490)/(I435 + I490).^[^ ^45^ ^]^

### Flow Cytometry Assay

The bacteria cultured overnight were diluted 100 times and expanded for 4 h, then centrifuged and resuspended with PBS, adjusting the OD_600_ to 0.5. Bacterial suspensions (10^6^ CFUs per mL) were cultured for 1 h at 37°C with SC (2 mg mL^−1^) and colistin (0.5 µg mL^−1^) alone or their combination. Then, the bacteria were mixed with PI (5 mm, 3 µL) and SYTO 9 (0.835 mm, 3 µL) and incubated for 15 min at room temperature in the dark. The CytExpert Flow Cytometer (Beckman, USA) measured ≈ 100 000 ungated events and analyzed the results using CytExpert 2.0 software.^[^ ^26^ ^]^

### Confocal Laser Scanning Microscopy

The sample pretreatment was the same as that of the Flow Cytometry Assay. After incubation at room temperature in the dark for 15 min, 20 µL bacterial suspension was dropped onto the slide, fixed with 4% glutaraldehyde, and observed with a CLSM microscope (Leica TCS SP2, Heidelberg, Germany).

### Measurement of ROS Levels

The level of ROS in *E. coli* G92 treated with colistin alone or colistin in combination with SFAs (1 mg mL^−1^) was measured using a fluorescent probe 2′,7′‐dichlorodihydro‐fluorescein diacetate (DCFH‐DA)^[^ ^46^ ^]^ (Beyotime, Shanghai, China). In short, the fluorescent probe DCFH‐DA (10 µm) was added to the bacterial culture and incubated at 37°C for 30 min, followed by washing twice with PBS. The bacterial mixture was treated with colistin alone or in combination with SFAs and incubated at 37°C for 1 h. With excitation and emission wavelengths of 488 and 525 nm, respectively, the fluorescence intensity was detected after 1 h of incubation.

### SOD Activity Measurement

The *E coli* G92 cultured overnight was expanded in a 1/100 ratio for 4 h. The bacterial cultures were centrifuged and resuspended with PBS to adjust an OD_600_ of 0.5, and then split into new 2 mL tubes. Bacterial suspension was treated for 4 h with colistin (0–8 µg mL^−1^), SFAs (0–4 mg mL^−1^) alone, or a combination of colistin (0–8 µg mL^−1^), and SFAs (1 mg mL^−1^). Intracellular superoxide dismutase (SOD) activity was measured using the Total Superoxide Dismutase Assay Kit with WST‐8 (S0101, Beyotime, China).

### Membrane Depolarization

To monitor the membrane potential, a fluorescent probe DiSC_3_(5) was used.^[^ ^47^ ^]^ In short, bacterial suspensions were incubated with DiSC_3_(5) (0.5 µm) for 30 min, and then colistin alone (0–8 µg mL^−1^) or in combination with SFAs (2 mg mL^−1^) was added for 1 h. With an excitation wavelength of 622 nm and an emission wavelength of 670 nm, the dissipated membrane potential of *E. coli* G92 was measured.

### ΔpH Measurement

Overnight *E. coli* G92 were resuspended to OD_600_ of 0.5 with PBS, and the final concentration of pH‐sensitive fluorescent probe BCECF‐AM^[^ ^48^ ^]^ (2 × 10^−6^ m) was added. Then, the bacterial mixture was treated with colistin (0.5 µg mL^−1^) or SFAs (2 mg mL^−1^) alone or in combination for 1 h at 37°C. With an excitation wavelength of 488 nm and an emission wavelength of 535 nm, the fluorescence intensity within 20 min was immediately determined.

### Bacterial PMF Detection

Briefly, 1 mL of bacterial suspension (OD_600_ = 0.6) was collected and diluted to 10^6^ CFU per mL. Then, the bacterial suspension was treated with CCCP, SC (2 mg mL^−1^), colistin (0.5 µg mL^−1^) alone or their combination at 37°C for 1 h. Next, the bacterial suspension was mixed with 10 µL of 3 mm DiOC_2_(3) (3,3′‐diethyl‐oxo‐iodocarbocyanine). The mixture was incubated at 37°C for 30 min. CytExpert Flow Cytometer (Beckman, USA) was used to measure signal intensity, and FlowJo V10.8.1 was used for analysis. Bandwidth band‐pass filter (488–530 nm) was used to detect green fluorescence, and a 488–610 nm bandwidth band‐pass filter was used to detect red fluorescence. The PMF was calculated and normalized as the ratio of the red/green fluorescence intensity. Membrane potential is calculated by the following formula: PMF = Lg (10^3/2^ × (red fluorescence/green fluorescence)).

### Swimming Motility Experiment

Agar media [0.3% (w/v)] composed of trypticase peptone (10 g L^−1^), NaCl (10 g L^−1^), and yeast extract (5 g L^−1^) was used to assess bacterial swimming motility.^[^ ^44^ ^]^ The colistin (0–8 µg mL^−1^) or SFAs (0–4 mg mL^−1^) alone or in combination with colistin (0–8 µg m^−1^) and SFAs (2 mg mL^−1^) were added after the medium reached 50°C. Each plate's center was filled with a 2 µL volume of *E. coli* G92 culture with an OD_600_ of 0.5, and it was left for 30 min. The swimming motility of bacteria was assessed by measuring the microsphere area after 48 h incubation at 37°C.

### Intracellular ATP Determination

The intracellular ATP concentrations of *E. coli* G92 were assessed using an Enhanced ATP Assay Kit (Beyotime, Shanghai). After treatment with colistin alone (0–8 µg mL^−1^) or in combination with SFAs (1 mg mL^−1^) for 4 h, bacterial cultures were centrifuged at 12 000 g at 4°C for 7 min, and the supernatant was removed. To measure the intracellular ATP levels, bacterial cells were lysed by precooled lysozyme, centrifuged, and the supernatant was collected. A 96‐well plate with the detecting solution added was incubated for 5 min at room temperature. The working solution for ATP detection was swiftly mixed with the supernatants before being applied to the wells. Intracellular ATP levels were determined from luminescence signals using the Infinite E Plex Microplate reader (Tecan).

### Efflux Pump Assay

The *E coli* G92 cultured overnight was expanded in a 1/100 ratio for 4 h. The bacterial cultures were centrifuged and resuspended with PBS to adjust an OD_600_ = 0.5. Subsequently, *E. coli* G92 was incubated with 5 × 10^−6^ m EtBr or known efflux pump inhibitor CCCP (1 × 10^−4^ m) at 37°C for 1 h. Then, the bacterial culture was treated with colistin (0.5 µg mL^−1^), SFAs (2 mg mL^−1^) alone, or a combination of them, and the fluorescence values at the excitation wavelength of 530 nm and emission wavelength of 600 nm were immediately measured within 30 min to characterize the EtBr content in the cells.

### Detection of Intracellular Accumulation of Colistin

The accumulated amount of colistin in *E. coli* G92 treated with different concentrations of SC was detected with the colistin enzyme‐linked immunosorbent assay (ELISA) kit. In short, *E. coli* G92 was diluted and expanded overnight for 4 h and resuspended with PBS and adjusted to OD_600_ = 0.6, and then divided into 2 mL tubes. Then, different concentrations of drugs were added to the bacterial culture and incubated for 1 h at 37°C. Follow the instructions to preprocess and dilute the sample for testing. 50 µL of sample or standard sample was taken into 96‐well plate, and then 50 µL of anti‐colistin antibody enzyme complex was added to culture. After five cleaning cycles, 50 µL of chromogenic solution was added and incubated at 37°C for 10 min, then the termination solution was added. The absorbance at 450 nm was measured. Results analysis and calculation were carried out according to the standard curve.

### Transcriptomic Analysis

*E. coli* G92 early‐exponential bacteria were treated with SC (0.25‐fold MIC) for 4 h at 37°C. After incubation, total RNA from the samples was extracted and quantified with a Nanodrop spectrophotometer from Thermo Scientific (MA, USA), and sequenced with an Illumina Hiseq 2000 system from Majorbio (Shanghai, China). Sequencing data were aligned with the reference genome for the following functional annotation analysis using GO and KEGG database. RSEM software^[^ ^49^ ^]^ was used to analyze the expression levels of genes quantitatively, and DESeq2^[^ ^50^ ^]^ was applied to identify the differentially expressed genes (DEGs).

### Determination of LPS Contents

Using the previously described chromogenic Limulus Amebocyte Lysate (LAL) test, the content of LPS in *E. coli* G92 treated with various doses of SC was evaluated.^[^ ^51^ ^]^ The *E. coli* G92 cultured overnight was expanded in a 1/100 ratio for 4 h. The bacterial cultures were centrifuged and resuspended with PBS to adjust OD_600_ = 0.5. Subsequently, *E. coli* G92 was treated with various doses of SC at 37°C for 4 h. Afterward, the sample was heated at 70°C for 15 min. Sample (50 µL) was placed into the wells. Each well received 50 µL of LAL reagent, and the mixture was then incubated at 37°C for 10 min. Each well was then filled with chromogenic substrate solution (100 µL, 2 mm), and the microtiter plate was incubated at 37°C for 6 min. The absorbance of suspension at 405 nm was determined after the enzymatic reaction was stopped by adding 50 µL of 25% acetic acid to each well. An *E. coli* endotoxin standard stock solution was used to create a standard curve that allowed the conversion of A_405nm_ readings into LPS concentrations.

### RT‐qPCR Analysis

Early‐exponential phase of *E. coli* G92 was exposed to colistin (0.5 µg mL^−1^) alone or in combination with SC (2 mg mL^−1^) for 4 h. After that, total RNA was extracted and measured using the 260/280 nm absorbance ratio. Using the PrimeScript RT reagent Kit with gDNA Eraser (Vazyme, Nanjing), reverse transcription of 1 µg of extracted RNA was carried out following the manufacturer's instructions. RT‐qPCR analysis was performed by 7500 Fast Real‐Time PCR System (Applied Biosystem, CA, USA) using the TB Green qPCR Kit (Takara) with the optimized primers (Table S3, Supporting Information). The fold changes in mRNA expression relative to the reference genes (16S rRNA) in *E. coli* were calculated using a relative quantitative technique.

### Western Blot Assays

Overnight *E. coli* G92 was resuspended to OD_600_ of 0.5 with PBS. After treatment with SC (0–2 mg mL^−1^) for 6 h, bacterial cells were collected to prepare western blot assay sample. The membrane was blocked, and incubated with primary antibody against MCR‐1 (prepared from mouse) and goat anti‐mouse IgG secondary antibody (HRP). Finally, the membrane was visualized with an enhanced chemiluminescence substrate and the blots were quantified using ImageJ software (GE Healthcare Life Sciences, UK).

### LPS Modification

According to a previous study,^[^ ^18^ ^]^ the proportion of LPS modification after treatment with different concentrations of SC was evaluated. Overnight cultured *E. coli* G92 was diluted with Phosphate‐Buffered Saline (PBS) at a ratio of 1:100, mixed with different concentrations of SC (0–4 mg mL^−1^), and incubated at 37°C for 4 h. The bacterial culture was analyzed by LC‐MS/MS after passing through 0.22 µm filter membrane.

### Determination of Membrane Charge

FITC‐labeled Poly‐L‐Lysine (PLL) was used to assess the charge of *E. coli* G92.^[^ ^52^ ^]^ First, *E. coli* G92 was treated with different concentrations of SC at 37°C for 4 h and washed with PBS. Then, bacterial suspension (300 µL) was incubated for 10 min at room temperature in a solution of Tris buffer (0.03 m, pH 8.0), 20% sucrose, and 20 µg mL^−1^ FITC‐PLL. Cells were then cleaned by three rounds of centrifugation and resuspension in Tris solution containing 20% sucrose to eliminate any unbound PLL. Black‐walled microtitre plate wells were added with 200 µL of spheroplast samples, and a microplate reader was used to measure the amount of FITC fluorescence (excitation at 490 nm and emission at 525 nm). The increase in fluorescence value represents a decrease in the positive charge on the surface of the cell membrane.

### Docking Analysis

A template was created using the crystal structure of the MCR‐1 protein (PDB accession number: 5GRR). MCR‐1 and SC were molecularly docked using the Autodock Vina tool without the use of water molecules. In a 2D graphic, Discovery Studio 4.5 was applied to depict the interactions of SC with the residues of the binding sites in MCR‐1.

### Site‐Directed Mutagenesis for MCR‐1

According to the results of molecular docking, the potential binding site of SC to MCR‐1 (Asp331, Gly334, Asp337) was mutated to alanine, respectively. Briefly, pET28a‐*mcr‐1* was used as a template. Primers for site‐directed residue mutagenesis of the expression vector (Table S4, Supporting Information) were used to construct the pET28a‐*mcr‐1* mutants (Asp331 Ala, Gly334 Ala, Asp337Ala). Afterward, the pET28a‐*mcr‐1* mutants were transformed into *E. coli* BL21 competent cells. The synergistic activity of SC and colistin against the mutants was determined by a checkerboard microdilution assay.

### *G. mellonella* Infection Model

*E. coli* G92 (10^7^ CFU per mL) suspension was applied to *G. mellonella* larvae (Huiyude Biotech Company, Tianjin, China) that were split into four groups (n = 8 per group). Group 1 received PBS treatment after 1 h post‐infection, groups 2 and 3 received colistin (1 mg kg^−1^) or SC (30 mg kg^−1^), and group 4 received colistin plus SC (1 + 30 mg kg^−1^). *G. mellonella* larvae survival rates were tracked for seven days.

### Ethical Statement

This study was performed according to the relevant guidelines of Jiangsu Laboratory Animal Welfare and Ethical of Jiangsu Administrative Committee of Laboratory Animals (SYXK‐2022‐0044). All animal experiments were approved by the Animal Care Committee of Yangzhou University.

### Mouse Peritonitis‐Sepsis Infection Model

*E. coli* G92 suspension (5.0 × 10^8^ CFUs) was intraperitoneally injected into female ICR mice that were split into four groups (n = 8 per group). Mice were given intraperitoneal injections of PBS, colistin (0.25 mg kg^−1^), SC (40 mg kg^−1^), or combinations of colistin and SC (0.25 + 40 mg kg^−1^) at 1 h after infection. For seven days, the survival rates and bacterial count in mice organs were monitored.

### Mouse Intestinal Infection Model

The female ICR Mice (divided into four groups of six in each group) were infected with a single dose of 5.0 × 10^8^ CFU *E. coli* G92 suspension through the intestinal tract. Mice were gavaged with PBS, colistin (1 mg kg^−1^), SC (30 mg kg^−1^), or the combinations of colistin and SC (1 + 30 mg kg^−1^) at 1 h after infection. After 24 h post‐infection, the bacterial loads in mice organs and feces were determined.

### Measurement of Inflammatory Factors and Histological Analysis

Liver tissue was collected and flushed in sterile PBS. Tumor necrosis factor‐α (TNF‐α), interleukin‐6 (IL‐6), interleukin‐1β (IL‐1β), and interleukin‐4 (IL‐4) concentrations were detected using corresponding ELISA kits (Beyotime, Shanghai) according to the manufacturer's instructions. The excised organs (liver, spleen, and kidney) were fixed in 4% paraformaldehyde and stained by H&E.

### Statistics and Reproducibility

GraphPad Prism version 9.0 was used to analyze all data, which was displayed as mean ± SD. One‐way or two‐way ANOVA for multiple comparisons was carried out following the in vitro testing. In the three mouse models, the significance of survival rates and bacterial loads were analyzed by the log‐rank (Mantel–Cox) test or the Mann–Whitney U test, respectively. The calculated *p* < 0.05 was defined as a significant difference (^*^ *p* < 0.05, ^**^ *p* < 0.01, ^***^ *p* < 0.001, and ^****^ *p* < 0.0001).

## Conflict of Interest

The authors declare no conflict of interest.

## Author Contributions

Y.L. and Z.W. designed and supervised the project. J.C. and J.S. performed experiments, analyzed data, and drafted the manuscript. C.C. helped perform experiments. M.H. helped analyze data. Y.L. and J.C. wrote and revised the manuscript. All of the authors read and approved the final manuscript.

## Supporting information

Supporting Information

## Data Availability

The data that support the findings of this study are available from the corresponding author upon reasonable request.
