# Supplementary material for: Structural‐Activity Relationship‐Inspired the Discovery of Saturated Fatty Acids as Novel Colistin Enhancers
Source: Adv Sci (Weinh). 2023 Aug 8;10(29):2302182. doi: 10.1002/advs.202302182 (PMC10582468; doi:10.1002/advs.202302182)
Supplement: Supplementary file 1 — Supporting Information [file ADVS-10-2302182-s001.pdf]

ADVANCED SCIENCE
Open Access

Supporting Information

for *Adv. Sci.*, DOI 10.1002/advs.202302182

Structural-Activity Relationship-Inspired the Discovery of Saturated Fatty Acids as Novel Colistin Enhancers

Jinju Cai, Jingru Shi, Chen Chen, Mengping He, Zhiqiang Wang and Yuan Liu**

ADVANCED SCIENCE
Open Access

Supporting Information

for *Adv. Sci.*, DOI 10.1002/advs.202302182

Structural-Activity Relationship-Inspired the Discovery of Saturated Fatty Acids as Novel Colistin Enhancers

Jinju Cai, Jingru Shi, Chen Chen, Mengping He, Zhiqiang Wang and Yuan Liu**

Supporting Information

Figures

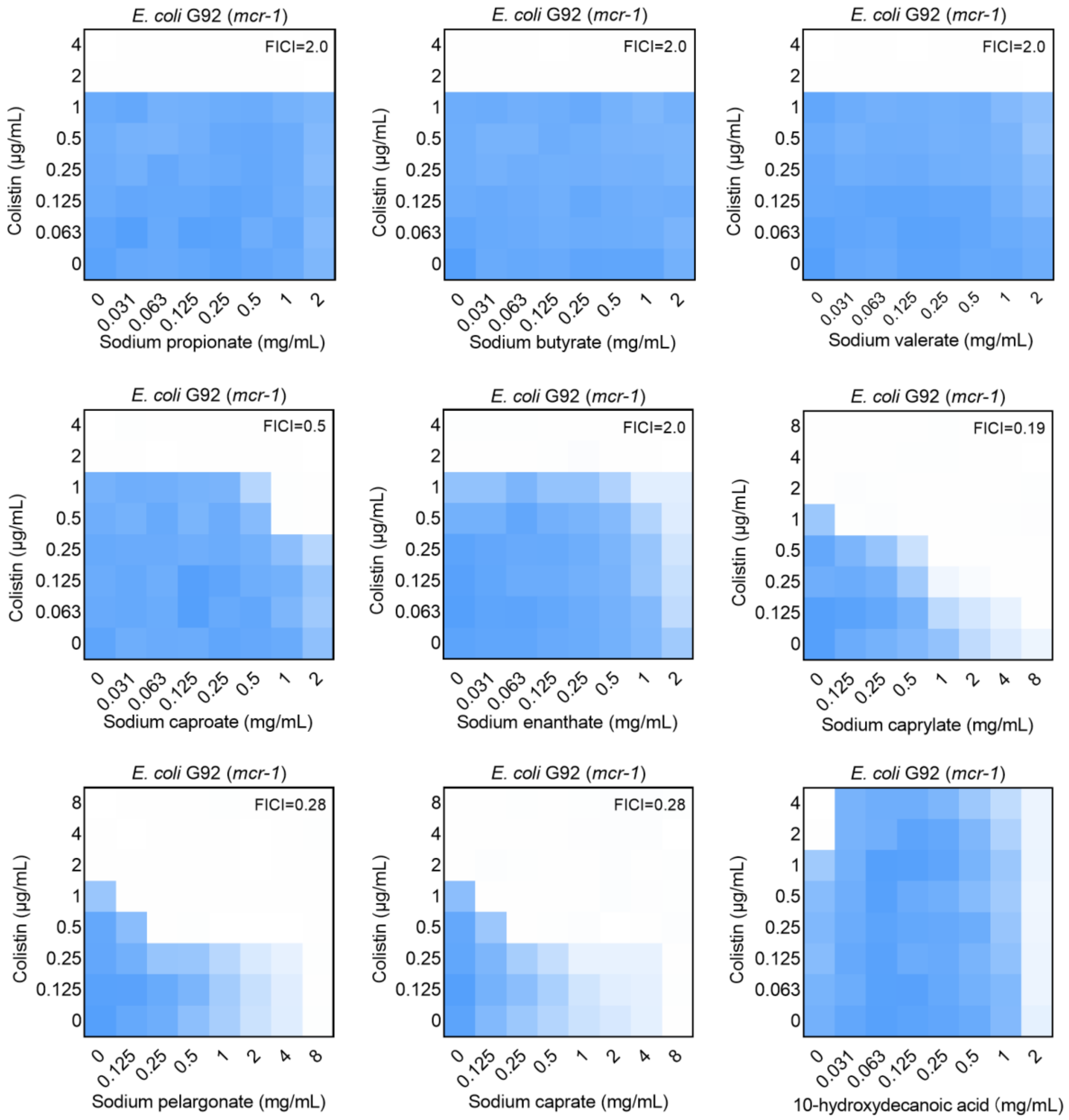

Figure S1. Checkerboard broth microdilution assays between nine SFAs and colistin against *mcr*-positive *E. coli* G92.

Dark blue regions represent higher bacterial cell density. The mean OD at 600 nm of biological replicates is shown.

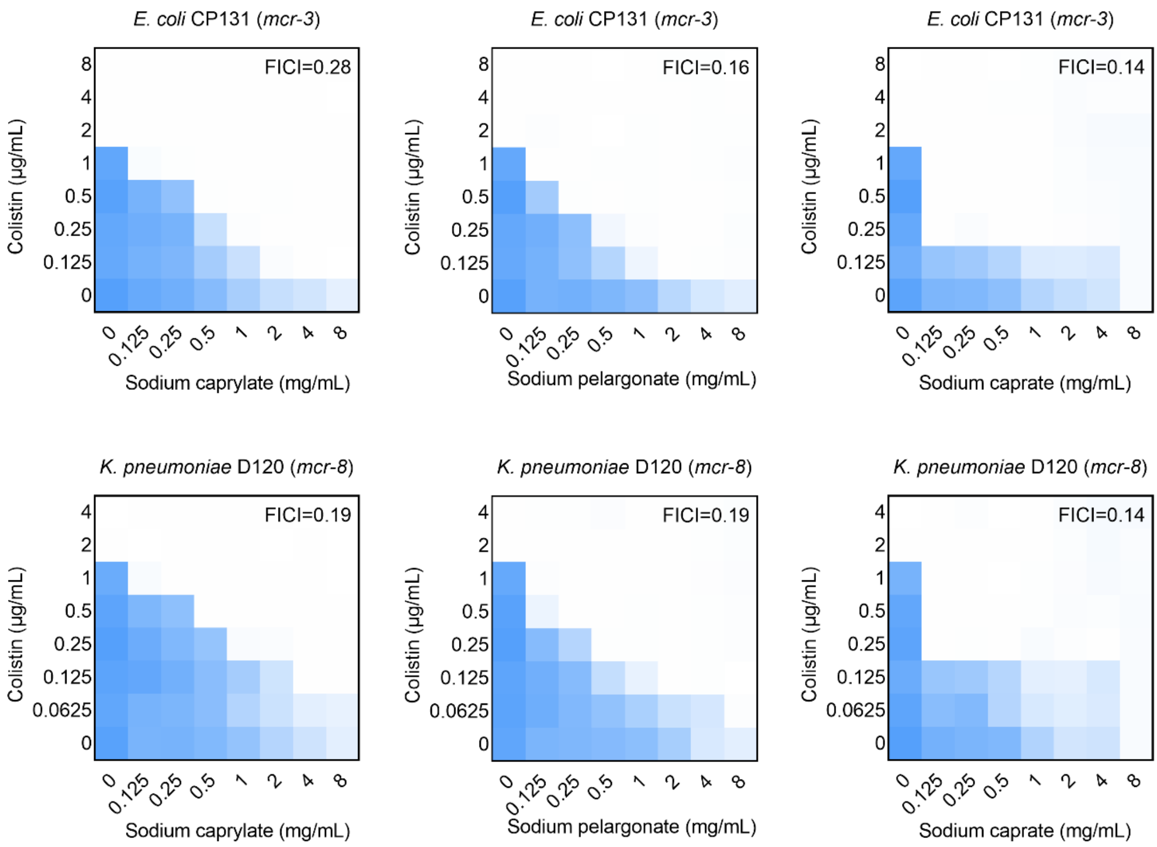

Figure S2. Checkerboard broth microdilution assays between three SFAs (SCL, SP and SC) and colistin against *mcr-3*/*mcr-8* positive *E. coli*/*K. pneumoniae*.

Dark blue regions represent higher bacterial cell density. The mean OD at 600 nm of biological replicates is shown.

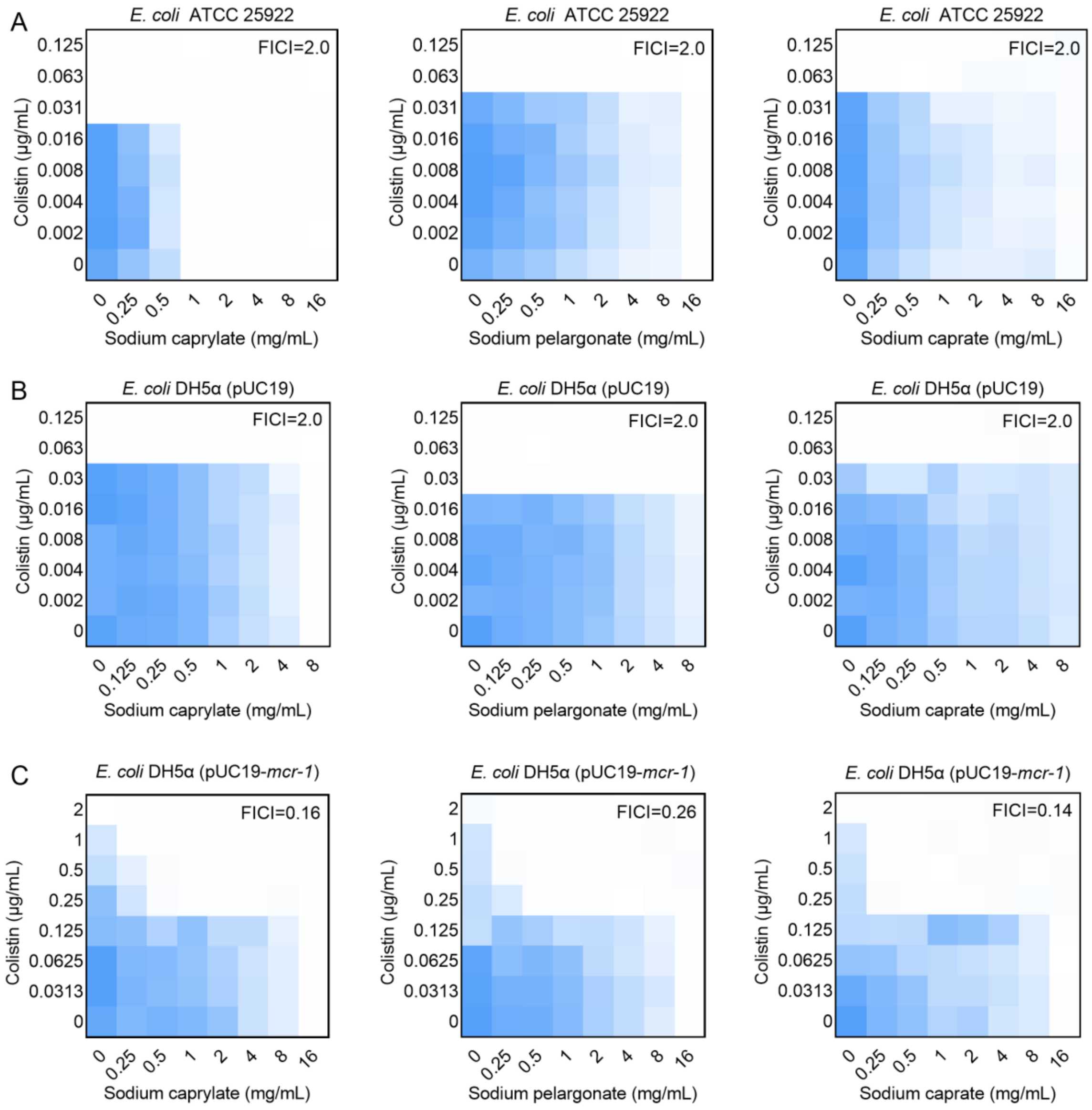

Figure S3. Synergistic effect of three SFAs (SCL, SP and SC) and colistin against drug-susceptible (A and B) and -resistant engineered bacteria (C).

Dark blue regions represent higher bacterial cell density. The mean OD at 600 nm of biological replicates is shown.

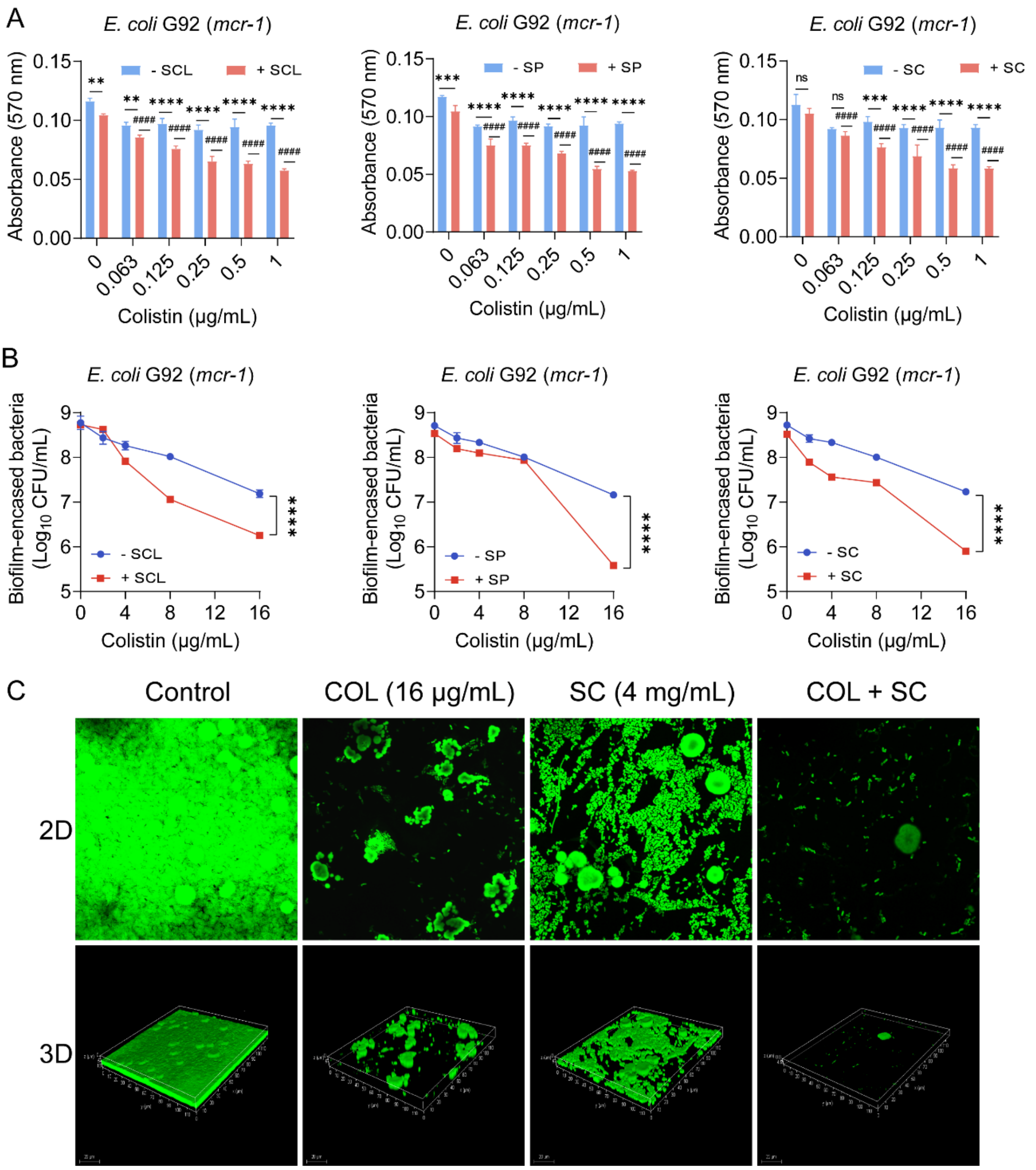

Figure S4. SFAs enhance the biofilm inhibition and eradication activity of colistin. **(A)** SFAs supplementation potentiate the inhibitory effect of colistin on *E. coli* G92 biofilm formation. **(B)** The addition of SFAs drastically promotes the eradication of established biofilm of *E. coli* G92 by colistin. **(C)** Confocal laser scanning microscopy image of the removal effect of established biofilm under single and combined treatment. Data were presented as mean ± SD from three biological replicates, and significance was determined by two-way ANOVA (n.s. indicates no significant difference, ***P* < 0.01, ****P* < 0.001, *****P* < 0.0001) or one-way ANOVA (colistin plus SFAs versus SFAs, $^{\#\#\#\#}P < 0.001$).

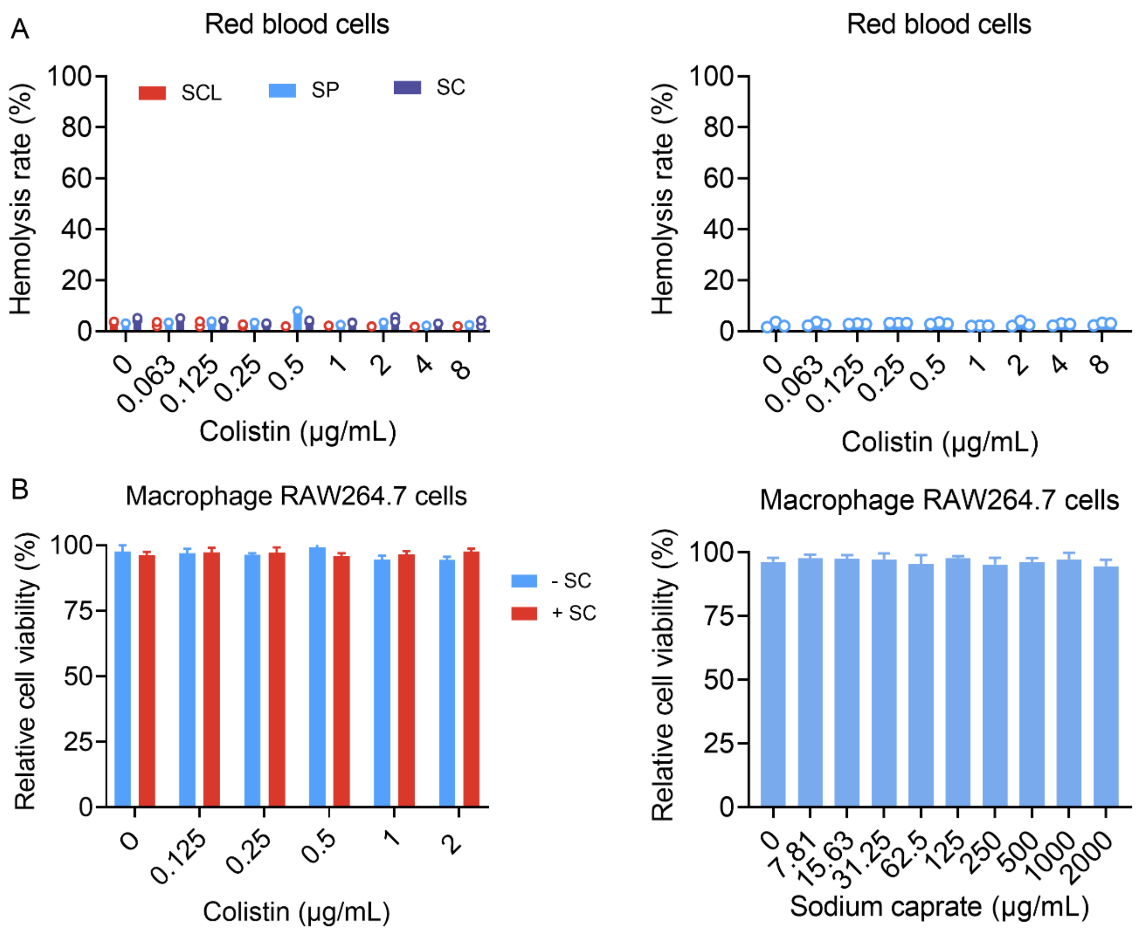

Figure S5. Safety evaluation of the combination of SFAs and colistin.

(A) Red blood cell hemolysis rate after treatment with different concentrations of colistin or combined with three SFAs (2 mg/mL). **(B)** Cytotoxicity analysis of different concentrations of colistin combined with SC (1 mg/mL) or different concentrations of SC used alone in RAW264.7 cells. Data were presented as mean ± SD from three biological replicates.

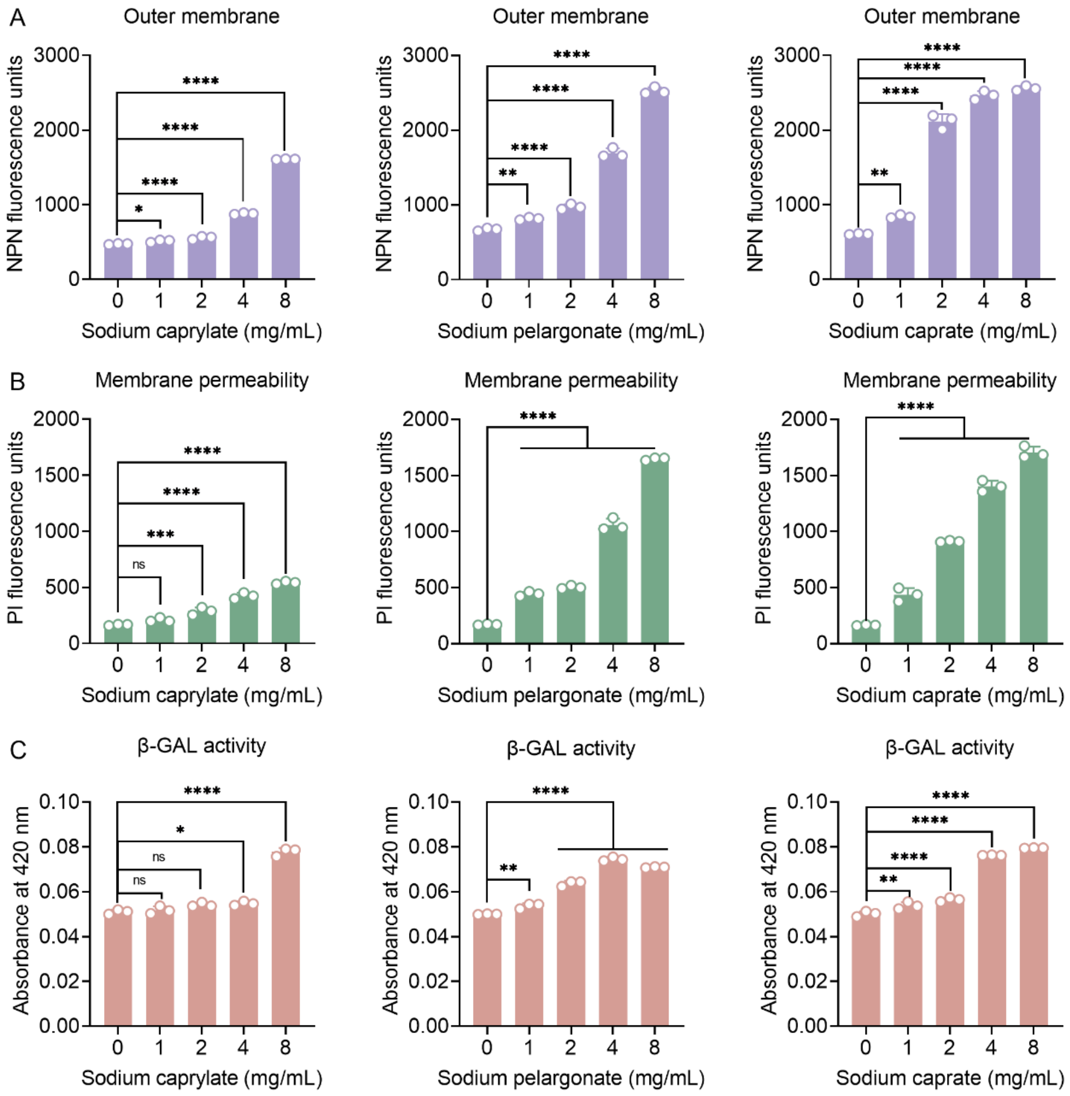

Figure S6. The effect of SFAs at sub-inhibitory concentrations on bacterial cell membrane.

(A and B) Outer membrane (OM) permeability (A) and integral membrane permeability (B) in *E. coli* G92 (*mcr-1*) in the presence of increasing concentrations of SFAs (0 to 8 mg/mL). OM permeability was evaluated by measuring the fluorescence intensity of *N*-phenyl-1-naphthylamine (NPN) after 1 h exposure to SFAs. Propidium iodide (PI) was used to measure integrity membrane permeability. **(C)** The activity of extracellular β-galactosidase after exposure to different concentrations of SFAs (0 to 8 mg/mL). Experiments were carried out with three biological replicates and data were given as mean ± SD. One-way ANOVA was used to evaluate statistical significance (**P* < 0.05, ***P* < 0.01, *****P* < 0.0001). ns, not significant.

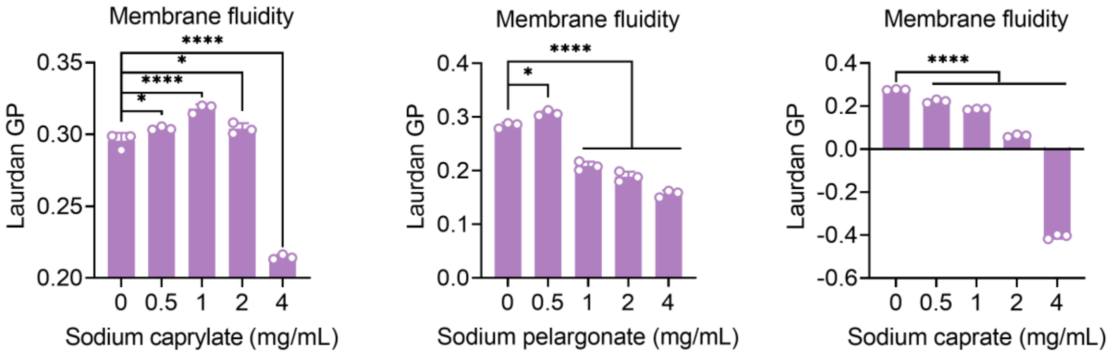

Figure S7. The addition of three SFAs enhances the fluidity of bacterial cell membrane. Membrane fluidity was determined using 10 μM Laurdan, and the fluorescence intensities were detected with emission wavelengths of 435 nm and 490 nm upon excitation at 350 nm. Experiments were carried out with three biological replicates and data were given as mean ± SD, and one-way ANOVA was used to determine statistical significance (**P* < 0.05, *****P* < 0.0001).

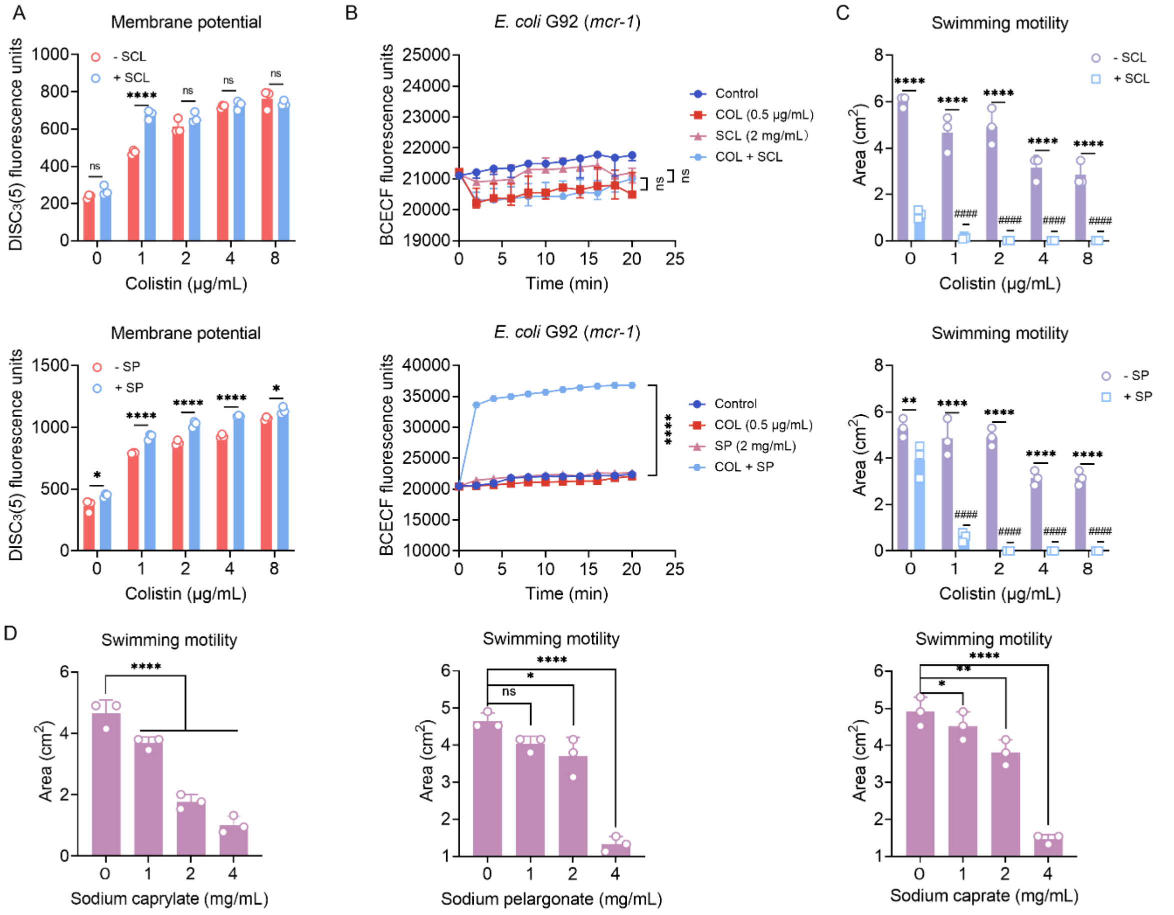

Figure S8. The impact of SCL or SP on bacterial proton motive force.

(A) Membrane potential changes of *E. coli* G92 after treatment with colistin alone or in combination with SCL or SP, determined by fluorescence probe DiSC$_3$(5). **(B)** ΔpH in BCECF-AM-labeled bacterial cells under combination of colistin and SCL or SP. Compared with the single use of colistin and SCL or SP, the fluorescence and cytoplasmic pH were up-regulated in combination group. **(C)** Swimming motility assay of *E. coli* G92 after treatment with colistin alone or in combination with SCL/SP. **(D)** Detection of swimming motility of *E. coli* G92 exposed to sub-MICs of SFAs. Overnight cultures were adjusted to OD_{600} of 0.5, and inoculated on 0.3% agar plates for 48 h at 37°C. Experiments were carried out with three biological replicates and data were given as mean ± SD, and one-way/two-way ANOVA was used to determine statistical significance (**P* < 0.05, ***P* < 0.01, *****P* < 0.0001). Statistical significance of colistin plus SFAs versus SFAs (1 mg/mL) was determined by one-way ANOVA, and shown as $^{####}P < 0.0001$.

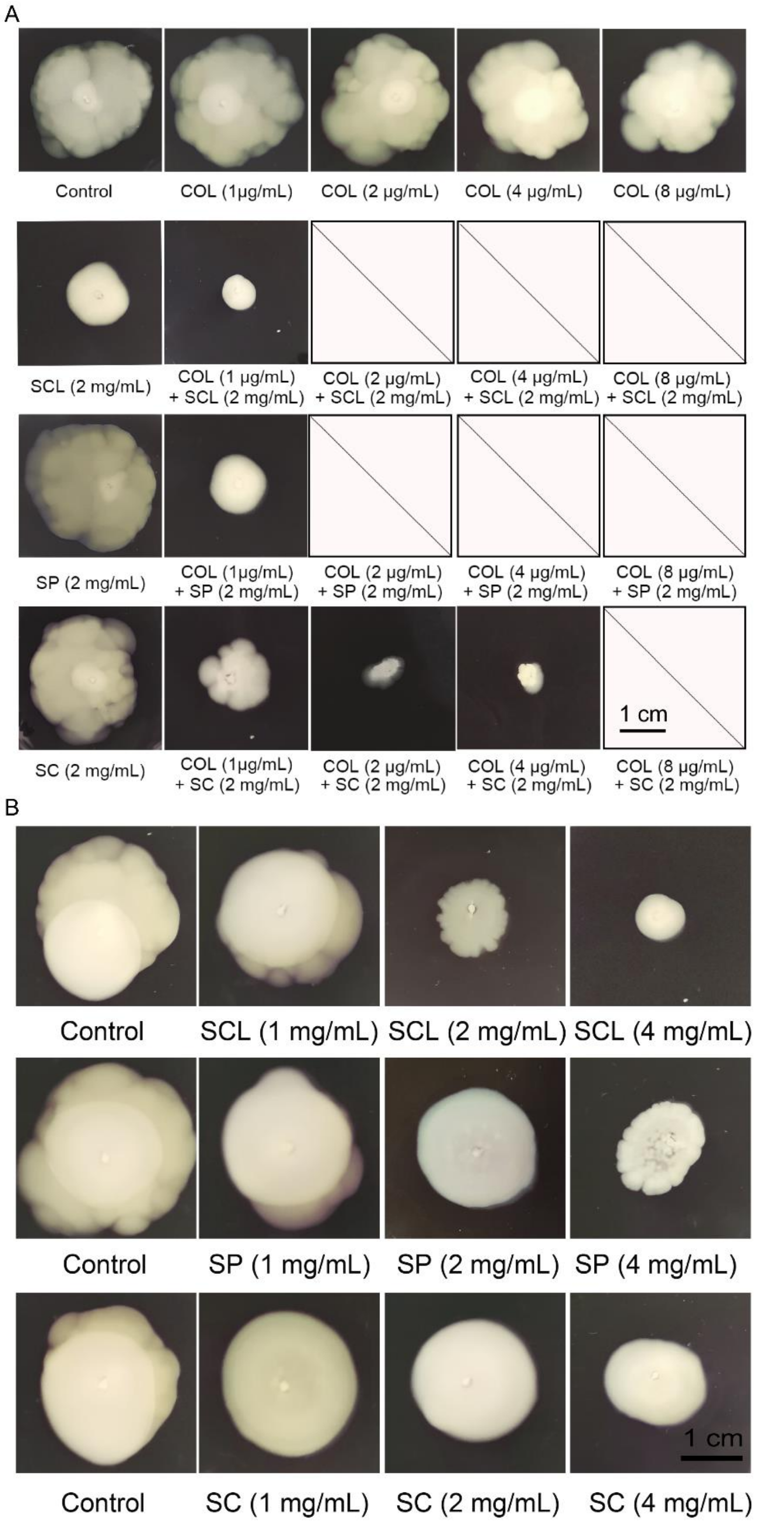

Figure S9. The effect of colistin combined with SFAs (A) or three sub-inhibitory concentrations of SFAs alone (B) on the swimming motility of *E. coli* G92.

0.3% agar media was applied to analyze bacterial swimming motility in the presence of different drugs. After incubation for 48 h, the size of the microsphere was measured and photographed. Scar bar, 1 cm. Experiments were carried out with three biological replicates.

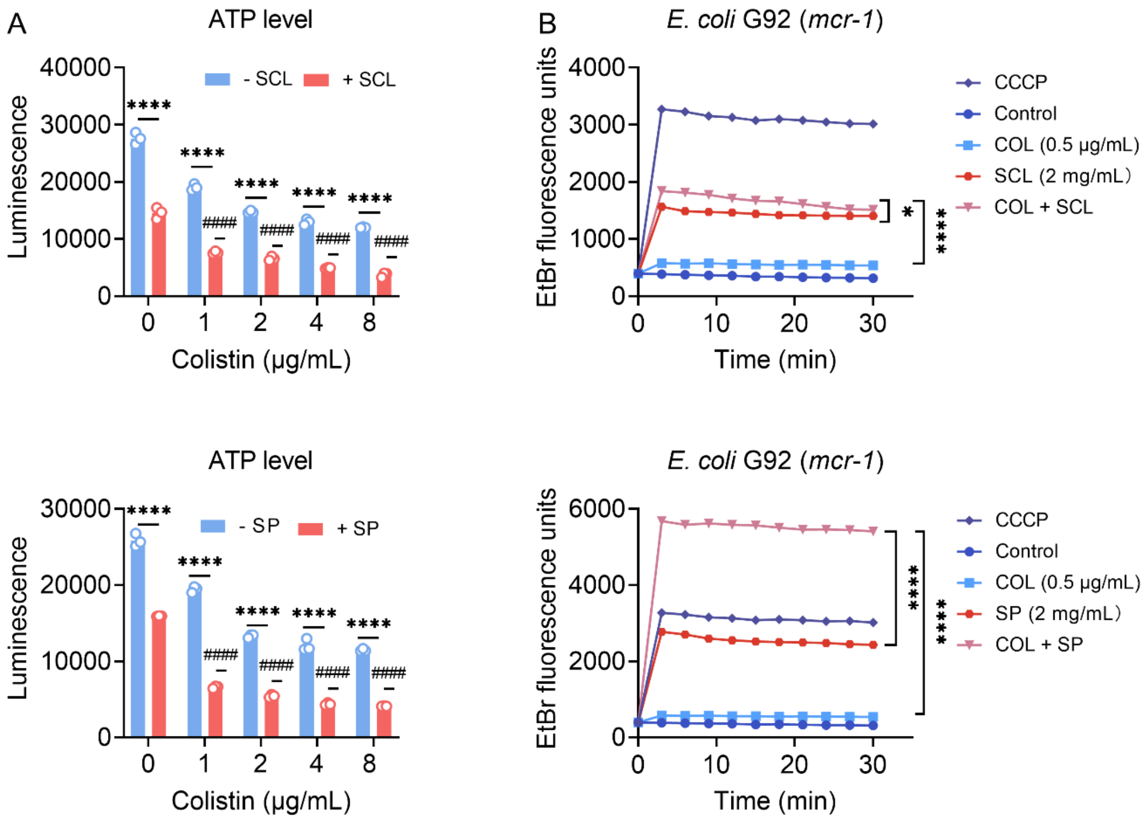

Figure S10. The effect of SCL or SP on ATP level and efflux pump function.

(A) Intracellular ATP production in *E. coli* G92 under the combination treatment compared with colistin alone. **(B)** Bacterial efflux pump activity in *E. coli* G92 using EtBr assay (λexcitation = 530 nm, λemission = 600 nm). Experiments were carried out with three biological replicates and data were given as mean ± SD, and one-way/two-way ANOVA was used to determine statistical significance (*$P < 0.05$, ****$P < 0.0001$) Statistical significance of colistin plus SFAs versus SFAs was determined by one-way ANOVA, and shown as $^{\#\#\#\#}P < 0.0001$.

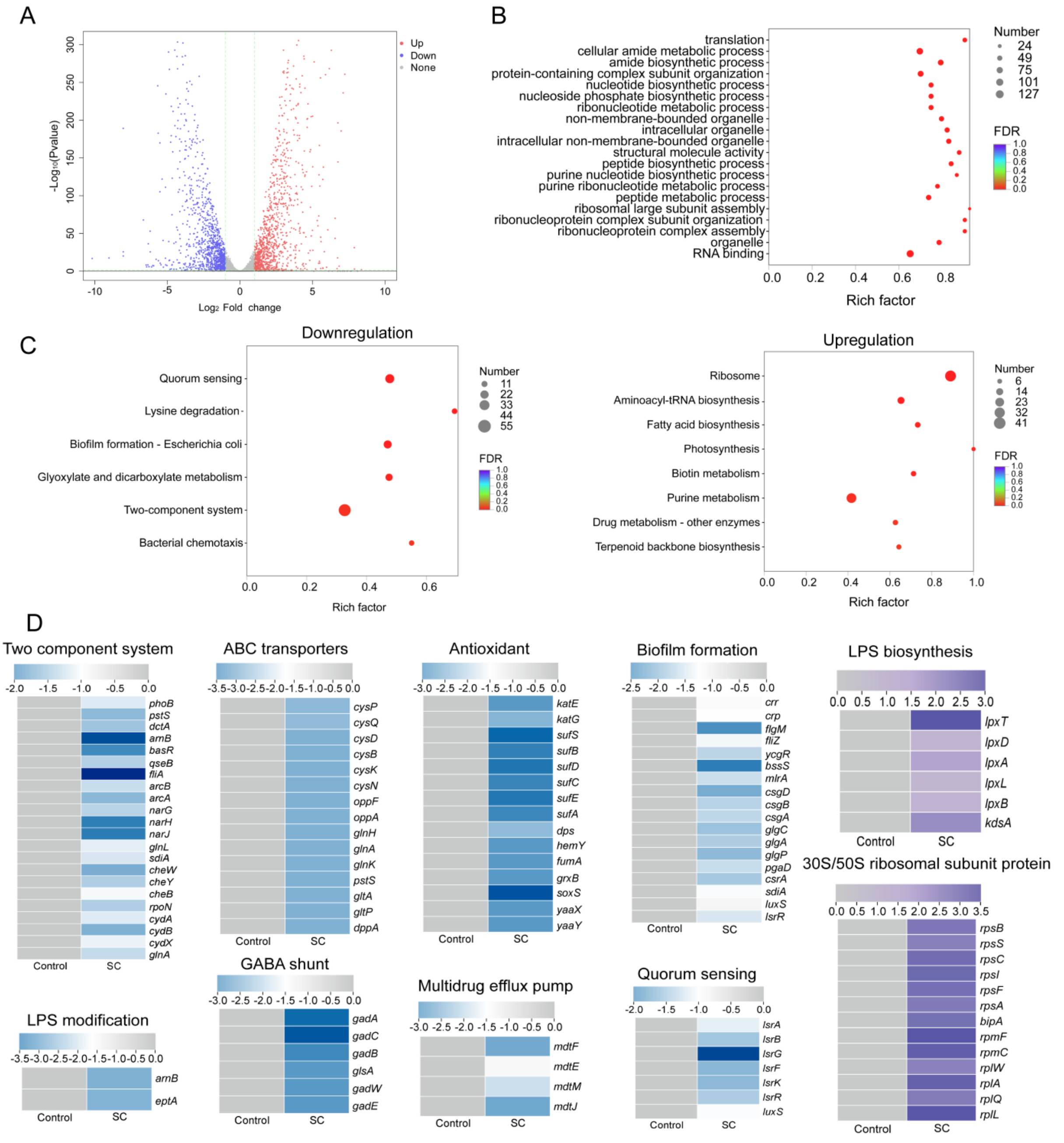

Figure S11. Transcriptomic analysis of *E. coli* G92 treated by SC.

Volcano plot **(A)** GO **(B)** and KEGG enrichment analysis **(C)** of the differential expression genes (DEGs) in *E. coli* G92 after exposure to PBS or SC. The x and y axis in A represent the expression changes and corresponding statistically significant degree, respectively. **(D)** Selected DEGs involved in two component system, ABC transporters, antioxidant reaction, biofilm formation, LPS biosynthesis, LPS modification, GABA shunt, multidrug efflux pump, quorum sensing, 30S/50S ribosomal subunit protein formation and other processes.

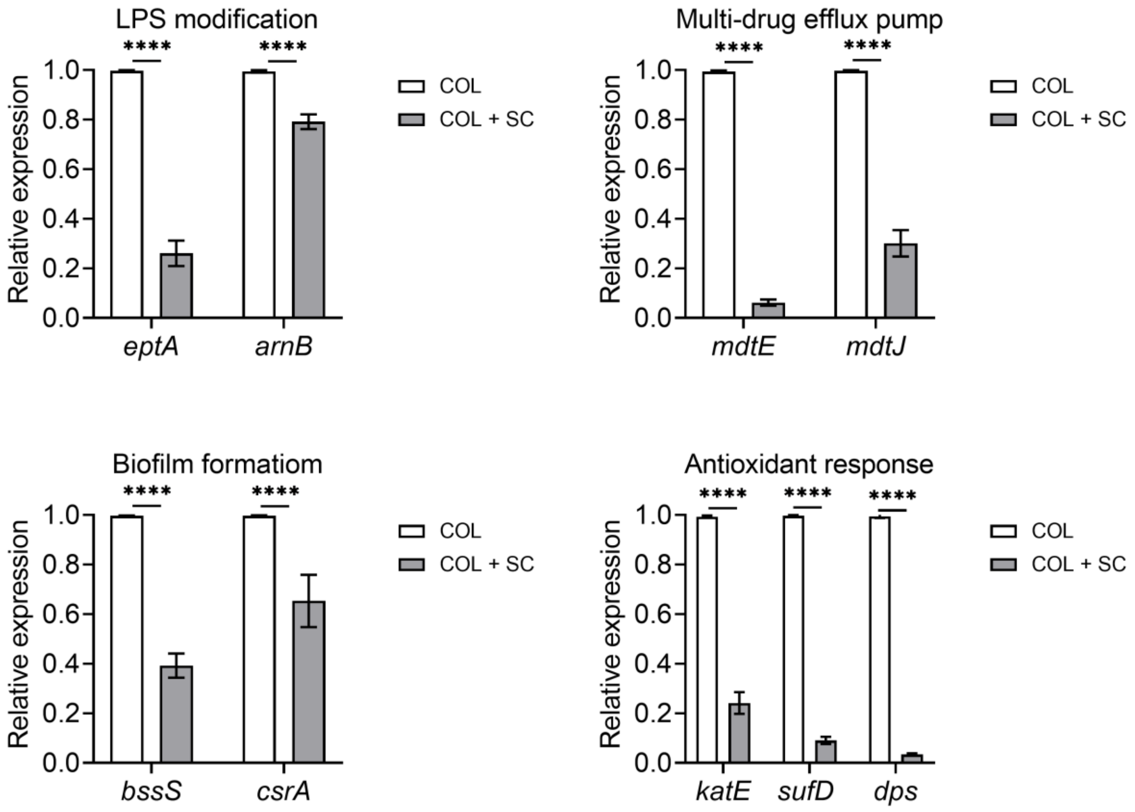

Figure S12. The effect of SC on the mRNA expression of representative genes in different pathways in *mcr-1*-positive bacteria.

RT-qPCR analysis of the expression of representative DEGs from transcriptome results. Significance was determined by an unpaired *t*-test (****$P < 0.0001$). 16S rRNA was used as a reference gene.

Tables

Table S1. The effect of various ions or serum on the synergistic activity of SFAs and colistin, as well as their antibacterial activity alone.

FICI	Control	+ Na^+	+ K^+	+ Mg^{2+}	+ EDTA	+ Serum
SCL	0.19	0.25	0.28	0.625	0.18	0.14
SP	0.28	0.31	0.28	0.53	0.14	0.05
SC	0.28	0.26	0.19	0.31	0.07	0.14
**MIC**	**Control**	**+ Na^+**	**+ K^+**	**+ Mg^{2+}**	**+ EDTA**	**+ Serum**
colistin (μg/mL)	2	2	2	4	2	2
SCL (mg/mL)	16	16	16	16	16	16
SP (mg/mL)	8	8	8	16	8	8
SC (mg/mL)	8	8	8	16	8	8

Table S2. Bacterial strains used in this study.

Strains	Sources
E. coli DH5α (pUC19)	In this study
E. coli DH5α (pUC19-*mcr-1*)	In this study
E. coli BL21 (pET28a-*mcr-1*)	In this study
E. coli G92 (*mcr-1*)	In this study
E. coli CP131 (*mcr-3*)	In this study
K. pneumoniae D126 (*mcr-8*)	In this study
E. coli DH5α (RP4-7)	In this study
E. coli EC600	In this study
E. coli LD67-1 (*mcr-1*)	In this study
E. coli LD93-1 (*mcr-1*)	In this study
E. coli ATCC 25922	In this study

Table S3. RT-qPCR primers used in this study.

Genes	Sequence (5’→3’)	Product (bp)
eptA	TTACAACCGCTATCCGCCTC	111
	ACCAGCGTGTTGTCGTAAGT	
arnB	CTGTTCTTTGCCGCTGTTCC	83
	ATTGTCCTGCGAGTTGCTGA	
mdtE	TTACGGGCGATTTTCCCCAA	88
	CATTCTGGCGGCTACCTTCA	
mdtJ	CTATTACGCTCTGCCCTCGG	117
	GGGGACAAATCCGCCAAGTA	
bssS	GTTTGCACTACCAGACCCCA	88
	TGTCTGGCAACATCAGTGGT	
csrA	AACCAGGTACGTATTGGCGT	77
	GCCTGGATACGCTGGTAGAT	
katE	CCGGAATACGAACTGGGCTT	125
	ATTTTGCCGACACGCTGAAC	
sufD	TCAATGGCGAAAACAGCACG	127
	GTTTTGTGCAACTGTCGGCT	
dps	ATCAACAGCAAAACCCCGCT	145
	CTGCGGTGTCGTCATCTTTC	
mcr-1	AAAGACGCGGTACAAGCAAC	213
	GCTGAACATACACGGCACAG	
16S rRNA	CCTACGGGAGGCAGCAG	194
	ATTACCGCGGCTGCTGG	

Table S4 The primer sequences of point mutations for MCR-1 protein.

Primers	Sequence (5’→3’)
MCR-1-ASP-331-Ala	AATAATTCGGCGTCAAAAGGCGTGATGGAT
	GCCTTTTGACGCCGAATTATTATCACGCCA
MCR-1-GLY-334-Ala	GACTCAAAAGCGGTGATGGATAAGCTGCCA
	ATCCATCACCGCTTTTGAGTCCGAATTATT
MCR-1-ASP-337-Ala	GCGTGATGGCGAAGCTGCCAAAAGCGCAA
	TGGCAGCTTCGCCATCACGCCTTTTGAGTC
